# Inducing a pH-dependent conformational response by competitive binding to Zn^2+^ of a series of chiral ligands of disparate basicity†

**DOI:** 10.1039/d1sc06812a

**Published:** 2022-01-17

**Authors:** Matthew M. Wootten, Bryden A. F. Le Bailly, Sofja Tshepelevitsh, Ivo Leito, Jonathan Clayden

**Affiliations:** School of Chemistry, University of Bristol Cantock's Close Bristol BS8 1TS UK j.clayden@bristol.ac.uk; Institute of Chemistry, University of Tartu Ravila 14a Tartu 50411 Estonia

## Abstract

Molecules that change shape in response to environmental conditions are central to biological molecular communication devices and their synthetic chemical analogues. Here we report a molecular system in which a series of chiral anionic ligands of differing basicity are selectively protonated according to the pH of the medium. A cationic circular dichroism (CD) reporter complex responds to anion binding by selecting one of two alternative enantiomeric conformations. Exploiting the principle that less basic anions have, in general, weaker electrostatic interactions than more basic anions, a set of three chiral acids with large (>5 unit) p*K*_a_ differences and differing configurations were sequentially deprotonated in acetonitrile by addition of base, allowing the most basic anion in the mixture at any time to bind to the reporter complex. A characteristic CD output resulted, which changed in sign as the next-most basic anion was revealed by the next deprotonation in the series. Four cycles of switching between three ligand-bound states were achieved with minimal changes in signal magnitude, by alternating addition of base and acid. The pH-dependent conformational response was used to transduce a signal by appending to the binding site a 2-aminoisobutyric acid (Aib) oligomer, whose *M* or *P* helical conformation depended on the chirality of the bound ligand, and was reported by a remote ^13^C-labelled NMR reporter group. The multicomponent system thus converts a pH signal into a programmable conformational response which induces a remote spectroscopic effect.

## Abstract 2

A chemical system comprising a series of chiral acids of differing p*K*_a_, a metal, and a conformationally labile chromophore responds to pH with a CD spectrum that reports the absolute stereochemistry of the most basic anion in the mixture.

## Graphical abstract

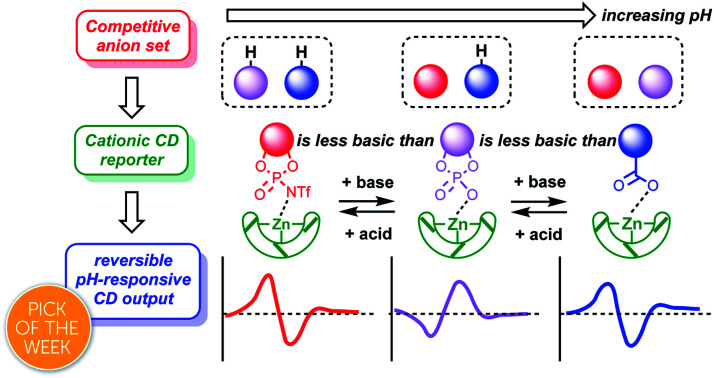

## Introduction

Signal transduction in biological systems is typically initiated by the non-covalent interaction between two or more biomolecules – categorised as a receptor and its ligand(s) – with a consequent change in conformation at the receptor translating that interaction into biochemical function by modulation of further intermolecular interactions.^1^ Biological receptors can function within the complex environment of the cell because selectivity is achieved by precise recognition of molecular features, allowing networks of signal transduction cascades to operate simultaneously and independently. The consequent ability of small molecules to act as selective agonists or antagonists of natural function through similar interactions with biomolecules forms the basis of pharmacological medicine.

Artificial chemical signal transduction systems, in which a chemical signal is detected through an intermolecular interaction and the resulting information is reported in the form of a spectroscopic (often colorimetric) output, are well established^2–5^ and form the basis of numerous diagnostic tests.^6–9^ Among the simplest and most well-known chemical signalling systems are pH indicators: interaction of the receptor molecule (the indicator) with a proton leads to a change in the receptor chromophore. ‘Universal’ indicators function through protonation of a series of indicators of increasing p*K*_a_, allowing the concentration of protons to be translated into a colorimetric output.^10^ Artificial signalling systems have been designed with biomimetic features such as spatial communication to remote sites,^11–13^ agonist/antagonist functions,^14,15^ and the ability to operate in the membrane phase.^16–19^

### Approach

We conceived of a way of combining the ability of a chemical system to respond to pH as an independent variable with the ability of a biomimetic system to translate environmental information into conformational change as a dependent consequence. Our approach was to use a suite of chiral acids with disparate p*K*_a_ values, having conjugate bases that may act as chiral ligands at a cationic metal site. This suite of acids translates information about pH into a chemical state: the acids of p*K*_a_ higher than local pH remain predominantly protonated, while those acids of p*K*_a_ lower than local pH are predominantly deprotonated to their anionic conjugate bases. The pH of the system is thus translated into a chemical state in which some acids are protonated and some are not. On a conceptual level the pH value is translated into a discrete output, defined by the number of acids that are deprotonated. This conversion of chemical inputs into multiple discrete outputs is reminiscent of molecular logic gates.^20,21^

So far, this concept is closely related to that of the universal indicator, but we needed a way to translate the chemical information about the protonation state of the suite of acids into a conformational response. We did this by employing a metal-containing receptor carrying a zinc cofactor as a binding site for the anionic conjugate bases. Key to the function of this system is the ability of a metal cation to interact selectively with the most basic of a series of competitive anions. According to hard–soft acid–base theory,^22^ hard–hard and soft–soft interactions between Lewis acids and bases are more favourable than hard–soft interactions. It follows that, given a mixture of anions, a hard Lewis acid receptor will preferentially bind the hardest Lewis base present. In the case of anionic centres of similar polarisability (as in our case – OH and NH acids) the hardness of an anion is broadly positively correlated with the p*K*_a_ of its conjugate acid. Thus, the anion with the highest p*K*_aH_, *i.e.* the most basic ligand, is typically the hardest.^23^ Upon addition of a sufficiently strong acid, this ligand becomes protonated and unable to bind to the receptor, allowing the next most basic ligand (with the second highest p*K*_aH_) to bind. In this way, a decrease of pH effects a ligand exchange. The use of pH to control the binding of multiple competitive ligands in this way remains unexplored; in our previous work, we demonstrated only pH-induced ligand exchange in non-metallic hydrogen-bonded systems.^15^

**Fig. 1:**
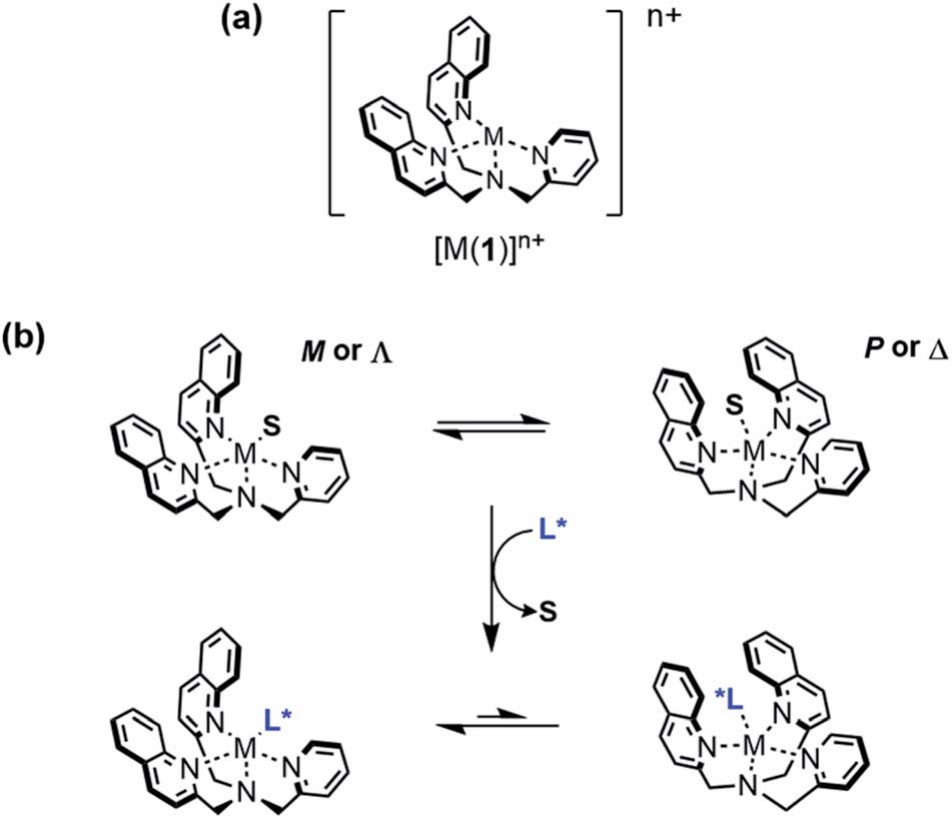
(a) Structure of [M(BQPA)]^*n*+^; (b) conformational preference induced by displacement of a solvent molecule S from the metal centre by a chiral ligand L*.

Circular dichroism (CD) spectroscopy is a well-established technique to probe conformational changes in supramolecular assemblies, and is commonly used to explore host-guest interactions.^24–26^ Typically, a response arises from a conformational change of the host induced by binding of the guest, but it is not necessary for the host to be chiral if a chiral guest can form a chiral host-guest complex. Canary and Anslyn *et al.* have developed transition metal complexes of *N*,*N*-bis(2-quinolyl)methyl-*N*-(2-pyridyl)methyl-amine (BQPA) 1 as versatile CD-reporting receptors.^27,28^ In solution, these propeller complexes rapidly interconvert between enantiomeric *P* (or Δ) and *M* (or Λ) conformations (Fig. 1). In the absence of a chiral bias, these conformations are populated equally, thus the overall CD signal is zero. However, coordination of a chiral guest can induce a preference for one conformation due to steric interaction between the ligands, giving a Cotton effect, which can be used as a CD output signal, at around 240 nm. These complexes have been used to determine the *ee* of scalemic mixtures of chiral carboxylic acids^27^ and differentiate between Boc-protected amino acids.^28^ More recently, we have reported metal(BQPA) analogues with pendent 2-aminoisobutyric acid (Aib) foldamers that can transmit stereochemical information from the metal binding site to a remote fluorescence^19^ or ^1^H-NMR^29^ reporter. An analogous metal binding Aib foldamer with a bis(pyridinyl)-triazolyl binding site has been shown by Webb *et al.* to form switchable ion channels that have antibacterial activity.^30^

We now report the use of pH to control the competitive binding of chiral anions to a receptor Zn(1)^2+^, using CD spectroscopy as a means of detecting which of the multiple ligands in the mixture is preferentially bound. By addition of acid or base to the system, the anionic ligands may be activated/deactivated sequentially, allowing reversible ligand exchange to translate the pH into a spectroscopic response in a programmable and relatively complex manner. We also show that an Aib foldamer appended to the receptor is capable of relaying information about the identity of the bound ligand to a remote spectroscopic reporter, providing proof of principle for the design of a complex pH-sensitive receptor–effector communication system.

## Results and discussion

### Identifying appropriate ligands

Previous work on these binding sites established that anionic ligands interact with the metal-(1) complex. Our proposal to use metal-(1) complexes to translate pH into conformational output requires a switchable response in which the anion interaction is turned on only in the presence of a sufficiently strong base. The conjugate acid of an anionic ligand is expected to interact only weakly with a metal-(1) complex as a result of reduced coulombic attraction. Treatment of a mixture of acid and metal-(1) complex with base should then induce a Cotton effect at 240 nm, characteristic of the anion's structure and absolute configuration, as deprotonation “unmasks” the active anionic ligand and allows it to bind to the metal centre.

We selected Zn(1)·2ClO_4_ as our receptor, using diamagnetic Zn^2+^ as the metal to allow use of NMR as an analytical tool. Acetonitrile (MeCN) was chosen as the solvent as it is sufficiently polar to dissolve ionic species, allows strong intramolecular hydrogen bonding to persist in Aib foldamers, and has a high autoprotolysis constant (p*K*_auto_ = 39).^31^ p*K*_auto_ represents the range of p*K*_a_ values of species that may exist in that solvent, so this allowed us to use anionic ligands with conjugate acids having a wider spread of p*K*_a_ values than in other polar solvents such as water (p*K*_auto_ = 14) or ethanol (p*K*_auto_ = 18.9).^32^*N*-Boc-d-prolinate 2^−^ is well established as a tightly binding carboxylate ligand capable of inducing strong Cotton effects with metal(1) complexes^28^ and was used as the ligand for our initial investigations.

**Fig. 2:**
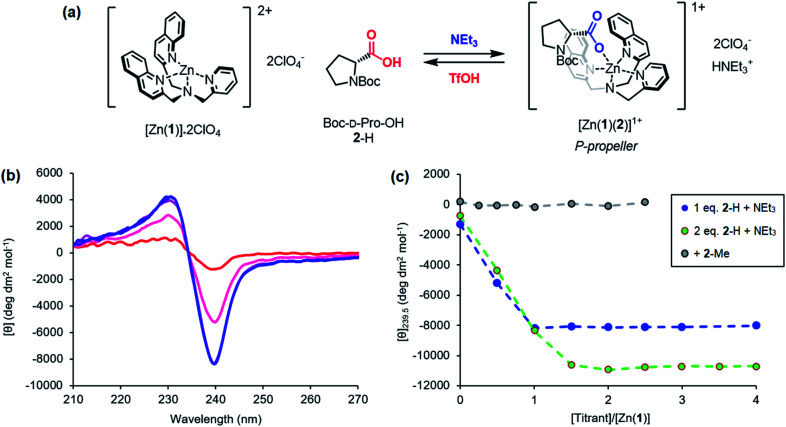
(a) Base-induced binding of 2-H; (b) CD spectra of Zn(1)·2ClO_4_ (*c*_0_ = 0.25 mM) with 2-H (1 eq.) in MeCN with addition of NEt_3_ (
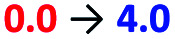
 eq.) in 0.5 eq. increments; (c) molar ellipticities at 239.5 nm of Zn(1)·2ClO_4_ (*c*_0_ = 0.25 mM) with (i) 2-H (1 eq.) and NEt_3_ (0.0 → 4.0 eq.), (ii) 2-H (2 eq.) and NEt_3_ (0.0 → 4.0 eq.), (iii) 2-Me (0 → 2.5 eq.).

Incremental addition of triethylamine to an equimolar 0.25 mM solution of Zn(1)·2ClO_4_ and 2-H in MeCN gave a negative Cotton effect at 239.5 nm indicative of a *P* propeller conformation,^27^ which reached a negative maximum of −8300 deg dm^2^ mol^−1^ at 1 equivalent of base (Fig. 2). This observation is consistent with deprotonation of 2-H leading to binding of its carboxylate conjugate base 2^−^ to the metal centre. Deprotonation of 2-H was mostly complete at this point, as further addition of triethylamine led to no significant change in signal strength. With two equivalents of 2-H, a negative maximum of −10 700 deg dm^2^ mol^−1^ was attained, indicating that not all zinc binding sites were occupied in the equimolar case. Both 2-H and 2^−^ had signals of approximately zero (see the ESI†), indicating that the observed output arose solely from the host complex. A small, negative signal was observed in the absence of base, suggesting that the neutral carboxylic acid is not ‘silent’, but still interacts weakly with Zn(1)^2+^. Since the methyl ester 2-Me gave no such “zero-base” signal when added to Zn(1)·2ClO_4_ (Fig. 2c), and the quinoline and pyridine groups of 1 are not basic enough to deprotonate 2-H to an appreciable amount (p*K*_aH_(MeCN) quinoline 11.97, pyridine 12.53 (ref. 33)), we ascribe this weak signal to hydrogen bonding between 2-H and 1.

Next, higher acidity ligands were investigated, with the intent to assess the relationship between ligand acidity and binding strength. BINOL-derived Brønsted acids are readily available as they are commonly used as organocatalysts,^34^ but these contain chromophores that would pose problems for interpretation of the CD spectra. Enantiomerically enriched binaphthyls display strong Cotton effects in the mid-ultraviolet region (200–300 nm),^35,36^ which would likely overlap with the Zn(1)^2+^ signal. However, TADDOL 3, which has also been utilised as a scaffold for chiral organocatalytic Brønsted acids,^37,38^ lacks these longer wavelength chromophores and was chosen as an alternative. Phosphoric acid 4-H and the more acidic *N*-triflylphosphoramide 5-H were prepared using opposite enantiomers of 3. Unfortunately, it was not possible to obtain a highly pure sample of phosphoramide 5-H. The leaching of metal cations during chromatographic purification of highly acidic compounds is a well-documented problem.^39–41^ A sample of 5-H was washed with 2 m HBr to remove these cations and reprotonate the phosphoramide anion, though each wash resulted in a ∼40% loss of material. The true concentration of a prepared phosphoramide stock solution was then calculated by titration with base, and then the volume of phosphoramide solution used in subsequent experiments was adjusted to the desired 1:1 stoichiometry of 5-H to Zn(1)·2ClO_4_.

**Fig. 3:**
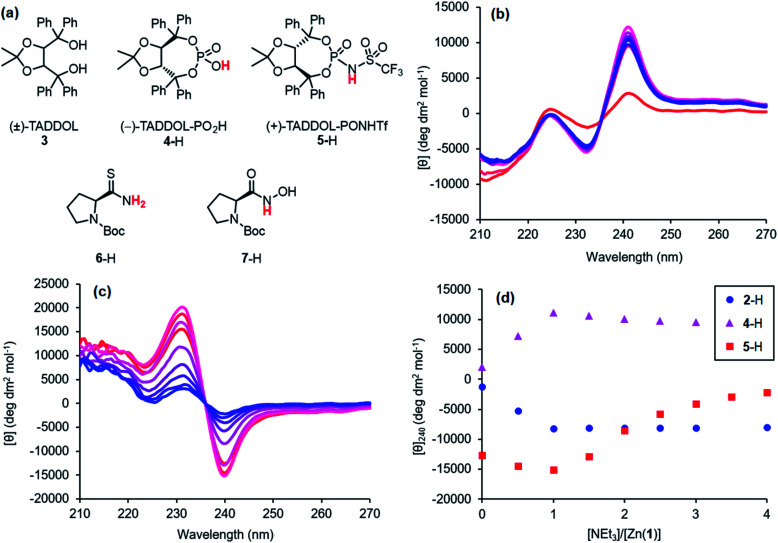
(a) Structures of 3 and ligands 4–7; CD spectra of Zn(1)·2ClO_4_ (*c*_0_ = 0.25 mM) with (b) 4-H (1 eq.) or (c) 5-H (1 eq.) in MeCN with addition of NEt_3_ (
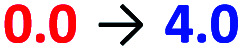
 eq.) in 0.5 eq. increments; (d) molar ellipticities at 240 nm of Zn(1)·2ClO_4_ (*c*_0_ = 0.25 mM) and 2-H, 4-H or 5-H (1 eq.) with addition of NEt_3_.

Addition of triethylamine to an equimolar 0.25 mM solution of Zn(1)·2ClO_4_ and either phosphoric acid 4-H or *N*-triflylphosphoramide 5-H induced strong Cotton effects at around 240 nm (Fig. 3b and c). The sign of the signal depended on the enantiomer of the parent diol; (−)-TADDOL-derived phosphate 4^−^ gave a positive signal while (+)-TADDOL-derived phosphoramide 5^−^ gave a negative signal. Although 4-H and 5-H displayed intrinsic Cotton effects, they were sufficiently separated from the Zn(1)^2+^ signal (see ESI†). Maximum signal strengths were achieved at 1 equivalent of triethylamine, indicating complete deprotonation. Excess triethylamine led to a reduction in signal strength, suggesting that the base may behave as a competitive ligand that begins to displace 4^−^ or 5^−^ from the metal centre as its concentration increases. The magnitude of the zero-base signal increased with the acidity of the protonated ligand *i.e.* its strength as a hydrogen bond donor (Fig. 3d). This further supports the hypothesis that the protonated ligands interact weakly with Zn(1)^2+^ through a hydrogen bonding interaction, since the more powerful hydrogen bond donors gave stronger signals while still requiring 1 equivalent of base to become fully deprotonated. In addition to these high-acidity ligands, the potential of ligands with acidities lower than 2-H was briefly investigated. Thioamide 6-H and hydroxamic acid 7-H derivatives of Boc-l-Pro-OH were prepared, but neither gave satisfactory results (see the ESI†).

**Fig. 4:**
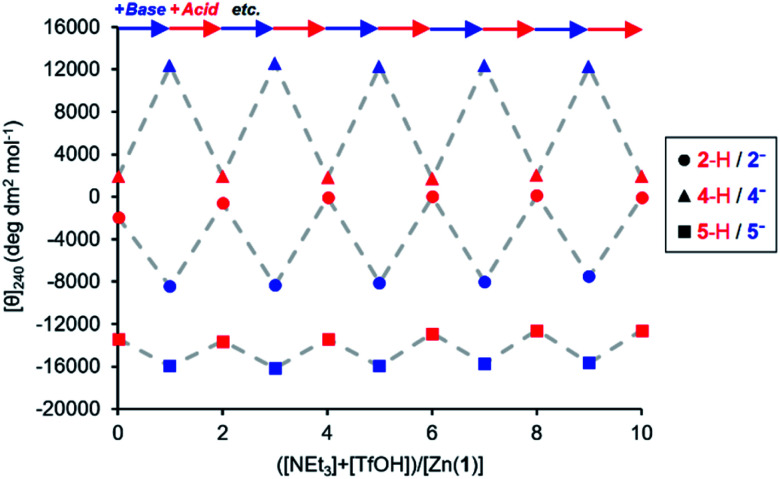
Molar ellipticities at 240 nm of Zn(1)·2ClO_4_ (*c*_0_ = 0.25 mM) with 2-H, 4-H or 5-H (1 eq.) in MeCN with alternating addition of NEt_3_ (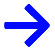) and TfOH (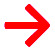) in 1 eq. increments.

The experiments described above show that anionic ligands may be activated *in situ* simply by deprotonating their conjugate acids. The corollary to this is that active ligands may be deactivated by protonation, and that the activation/deactivation cycle should be reversible. For each ligand, an equimolar 0.25 mM solution of Zn(1)·2ClO_4_ and ligand in MeCN was alternately treated with triethylamine (1 eq.) and then trifluoromethanesulfonic acid, TfOH (1 eq.) and the CD signal at 240 nm recorded after each addition (Fig. 4). As expected, in all cases addition of base gave a large increase in signal magnitude due to ligand activation by deprotonation, while subsequent addition of acid returned the system to its original state, giving a large decrease in signal magnitude. These single-ligand systems were robust, with very little variation in the signal magnitudes of the on and off states over five base/acid cycles.

### Quantifying ligand p*K*_a_ values

The p*K*_a_ values for all ligands were estimated computationally using the COSMO-RS method^42^ with empirical corrections (Table 1). For our purposes, these approximate values were sufficient to assess the differences in p*K*_a_ for the ligands. The three best-performing ligands (2-H, 4-H and 5-H) had excellent separation (>5 units) in p*K*_a_ values, indicating that a mixture of these acids could be deprotonated sequentially and asynchronously. Surprisingly, the p*K*_a_ calculated for 2-H (22.6 ± 1.5) was several units higher than the p*K*_aH_ of triethylamine (p*K*_aH_(MeCN) = 18.83 (ref. 33)). This suggests that very little deprotonation should occur with this acid–base pair, contrary to our observation of near-complete deprotonation with just 1 eq. of base. 2-H therefore has a lower apparent p*K*_a_ in our system.

**Table 1:** Estimated and reported p*K*_a_ values for acids/bases used in this study

Acid	Base	p*K*_a_
6-H	6^−^	29 ± 1^a^
[^t^BuP_1_(dma)_3_-H]^+^	^t^BuP_1_(dma)_3_	26.98^b^
7-H	7^−^	24.5 ± 1.0^a^
2-H	2^−^	22.6 ± 1.5^a^
[HNEt_3_]^+^	NEt_3_	18.83^b^
4-H	4^−^	17.0 ± 1.5^a^
5-H	5^−^	8.5 ± 0.8^a^
TfOH (CF_3_SO_2_OH)	TfO^−^	2.60^c^

a Estimated using COSMO-RS.

b Reported values from ref. 33.

c Reported values from ref. 32.

Several factors may contribute to a lower apparent p*K*_a_. Firstly, trace amounts of water are known to lower the p*K*_a_ of acids in non-aqueous solvents by stabilising the conjugate anion, which is otherwise poorly solvated by bulk acetonitrile.^43,44^ Secondly, carboxylates can form hydrogen-bonded complexes with their conjugate acids in acetonitrile, a process known as homoconjugation or homoassociation.^45^ The anionic conjugate base is stabilised by hydrogen bonding, so the apparent p*K*_a_ is decreased. However, given that 2-H was deprotonated quantitatively, homoconjugation is unlikely to be important in this system; such stabilisation takes place only until half of the acid is deprotonated. While deprotonating the remaining half, homoconjugation hinders deprotonation, because the neutral acid available in solution is stabilised by hydrogen bonding to the anion. Thirdly, and perhaps most importantly, the carboxylate is also stabilised by coordination to the divalent zinc complex:1
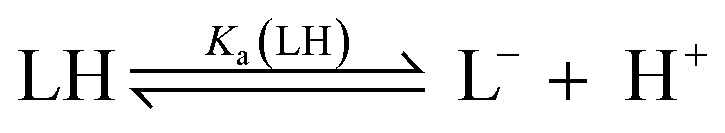
2
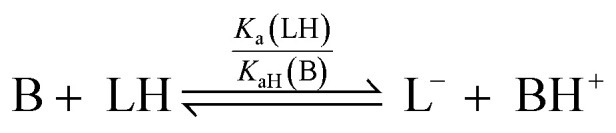
3
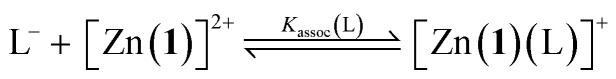
4
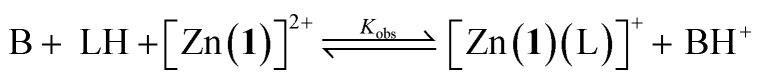


For a simple acid–base equilibrium between an arbitrary neutral base B and protonated ligand LH (eqn (2)), the equilibrium constant is simply the quotient of acidity constants *K*_a_(LH)/*K*_aH_(B), which are defined by eqn (1). In the presence of the zinc complex (eqn (4)), the equilibrium constant takes a slightly more complex form:5
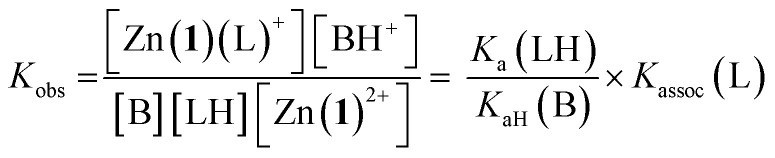
where *K*_assoc_(L) is the association constant for ligand L defined by eqn (3). Note that we have made the approximation that Zn(1)^2+^ binds only one ligand for simplicity. Comparing the equilibrium constant for eqn (2) and *K*_obs_ (eqn (5)) we see that *K*_obs_ is larger by a factor of *K*_assoc_(L). Addition of Zn(1)^2+^ to an acid–base equilibrium thus shifts the equilibrium towards the right hand side *i.e.* a greater proportion of ligand becomes deprotonated and the ligand appears to have increased in acidity. The apparent acidity constant *K*_a,app_(LH) can therefore be defined as product of the observed overall equilibrium constant *K*_obs_ and the acidity constant of the protonated base *K*_aH_(B) (eqn (6)):

6 *K*_a,app_(LH) = *K*_obs_ × *K*_aH_(B)

7 *K*_a,app_(LH) = *K*_a_(LH) × *K*_assoc_(L)

The consequence of eqn (7) is that any ligand with favourable binding with Zn(1)^2+^ (*i.e. K*_assoc_ > 1) will, under our experimental conditions, appear to be more acidic than its ‘true’ p*K*_a_ would suggest.

### Quantifying ligand association constants

**Fig. 5:**
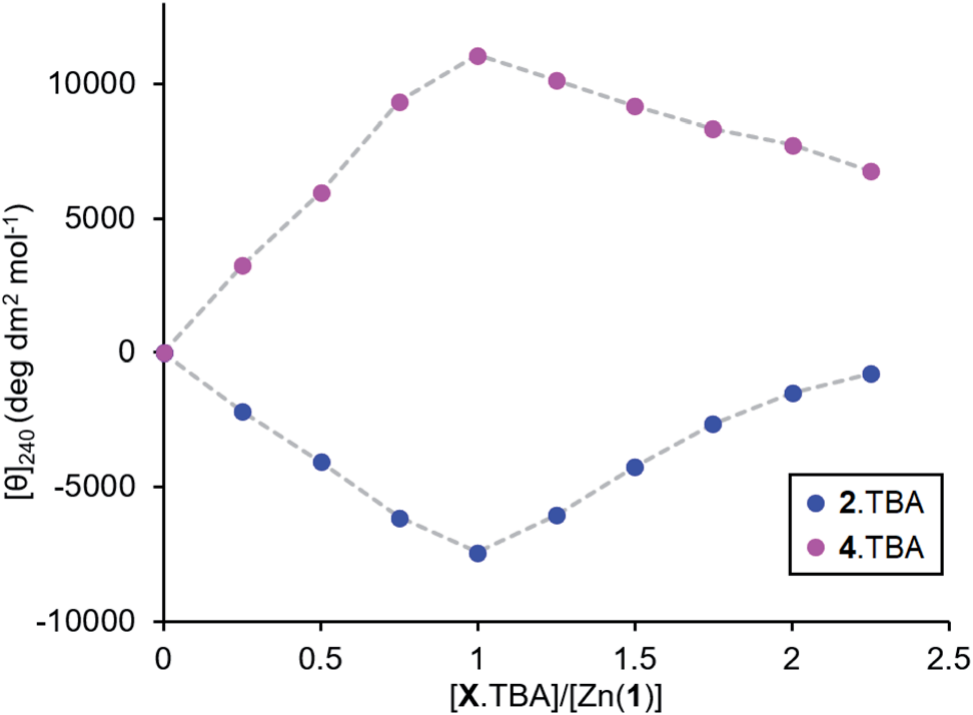
Molar ellipticities at 240 nm for Zn(1)·2ClO_4_ (*c*_0_ = 0.25 mM) in MeCN with addition of 2.TBA or 4.TBA (0 → 2.25 eq.).

Measuring the association constants by titrating a 0.25 mM MeCN solution of Zn(1)·2ClO_4_ with tetrabutylammonium (TBA) salts of 2-H and 4-H proved to be challenging. In both cases, excess ligand caused the signal to decrease rapidly, suggesting that multiple ligands bind to the Zn centre (Fig. 5). The TBA cation offers poor stabilisation for anions as its positive charge is shielded by large *n*-butyl groups and cannot act as a hydrogen bond donor. Therefore, the most energetically favourable configuration is for excess ligand to displace 1 (either in whole or in part) and bind to a Zn centre which already has a ligand bound. Multiple ligand binding was not observed with 2^−^ and triethylammonium as the cation (Fig. 2c, above) – the mildly acidic triethylammonium cation is able to stabilise anions through hydrogen bonding, so excess ligand may remain in bulk acetonitrile instead of displacing 1.

The very straight V-shaped binding curves suggested that the association constants were too large for CD spectroscopy to be an appropriate analytical method;^46^ attempts to fit the data to a 2:1 binding model failed to produce association constants with acceptable error values. Nevertheless, the behaviour of the single ligand systems in the excess-base regime (Fig. 3d, above) gives a qualitative measure of the relative binding strengths.

Upon addition of excess triethylamine, the carboxylate 2^−^ signal remained constant, while the phosphate 4^−^ and phosphoramide 5^−^ signals decreased in magnitude with a more pronounced decrease for the phosphoramide. This was ascribed to displacement of the ligand from the metal centre by the base. The least basic phosphoramide ligand was more easily displaced than the phosphate, which was in turn more easily displaced than the most basic carboxylate ligand. The association constants for the ligands therefore follow the order *K*_assoc_(phosphoramide) < *K*_assoc_(phosphate) < *K*_assoc_(carboxylate). Since the negative charge is dispersed to a greater extent in anions with lower p*K*_aH_ values (*i.e.* less basic ligands), we expect that the coulombic attraction between such anions and the cationic metal centre must be weaker. There are, of course, both steric (from the chiral scaffold) and covalent contributions (from the ligand lone pairs) to the overall binding strength in addition to coulombic attraction, but to a first approximation we observe that *K*_assoc_ is negatively correlated with *K*_a_.

### Conformational switching with multiple competitive ligands

Given an equimolar mixture of ligands, the Zn(1)^2+^ receptor will preferentially bind with the ligand with largest association constant. We therefore envisaged that conformational preference in the receptor could be selectively induced simply by controlling the pH in a multiple-ligand system, with the positive or negative Cotton effect reporting the conformational response. Titrating base into a mixture of acids leads first to deprotonation of the ligand with the lowest p*K*_aH_, which will induce a Cotton effect as it binds to Zn(1)^2+^. Adding further base deprotonates the ligand with the next lowest p*K*_aH_,^47^ which should displace the first ligand from the metal centre, since *K*_assoc_ is negatively correlated with *K*_a_. If the two ligands induce Cotton effects of opposite sign, then this pH-induced ligand exchange should be observable by CD spectroscopy.

**Fig. 6:**
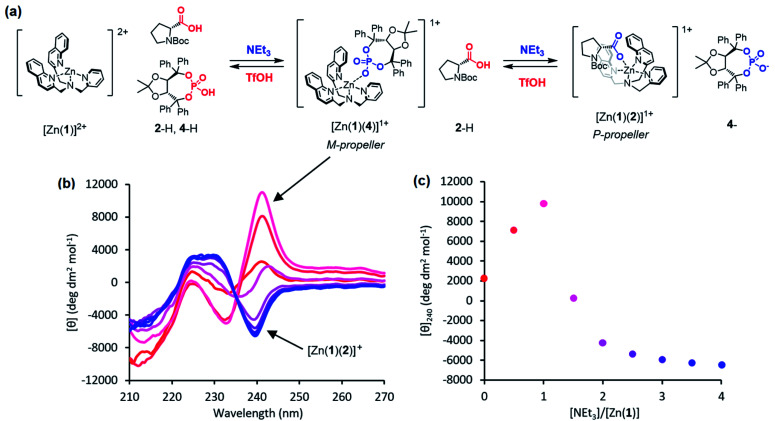
(a) Base-induced binding of 4-H followed by displacement by 2^−^ (triethylammonium and triflate spectator anions omitted for clarity); (b) CD spectra of Zn(1)·2ClO_4_ (*c*_0_ = 0.25 mM) with 2-H (1 eq.) and 4-H (1 eq.) in MeCN with addition of NEt_3_ (
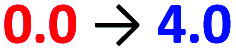
 eq.) in 0.5 eq. increments; (c) molar ellipticity at 240 nm for the same system.

Exploiting their favourable p*K*_a_ values and association constant data, we first tested a two-ligand exchange with 2-H and 4-H. As the first equivalent of triethylamine was added to a solution of equimolar 0.25 mM concentrations of each of 2-H, 4-H and Zn(1)·2ClO_4_, a positive signal was induced, indicating that the more acidic phosphoric acid was deprotonated first, as expected (Fig. 6). Further addition of base caused the signal to become negative, as the base deprotonated 2-H, whose anion then displaced 4^−^ from the metal centre. Deprotonation of 2-H was not complete after addition of the second equivalent of triethylamine; the CD signal continued to decrease with addition of a further 2 eq. of base, suggesting that 2-H has a higher apparent p*K*_a_ than in the single ligand case. The carboxylate signal levelled off at *ca.* −6500 deg dm^2^ mol^−1^, a lower value than in the single acid system. The increase in the apparent p*K*_a_ of 2-H may be rationalised by considering the equilibrium constant for a general base-induced ligand exchange (eqn (8)):8
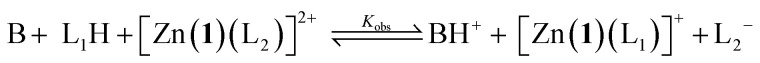
9
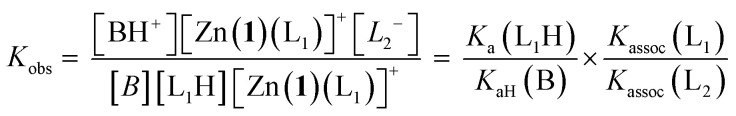
10
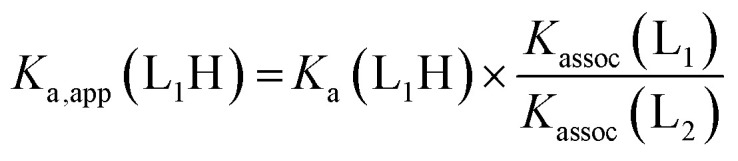
where L_1_^−^ is the more basic carboxylate ligand 2^−^ and L_2_^−^ is the less basic phosphate ligand 4^−^ in this case. Again, we make the approximation that Zn(1)^2+^ only binds one ligand. Using the definition of *K*_a,app_ (eqn (6)) with *K*_obs_ (eqn (9)), we see that the apparent acidity of the carboxylic acid *K*_a,app_(2-H) (eqn (10)) is reduced by a factor of *K*_assoc_(4^−^), the association constant of the phosphate ligand, with respect to the single ligand system (eqn (7)). In chemical terms, the phosphate ligand blocks the Zn(1)^2+^ binding site, making it less available to stabilise the carboxylate anion. Reduced anion stabilisation decreases the acidity of the carboxylic acid. The lower-than-expected carboxylate signal may have been caused by ligand competition. Incomplete displacement of the phosphate by the carboxylate leaves a small amount of phosphate-bound Zn(1). The CD signal of this phosphate-bound Zn(1) would partially cancel that of the carboxylate, leading to an overall reduction in signal magnitude. Alternatively, a higher-coordination species with both ligands bound to the metal centre may be formed.

**Fig. 7:**
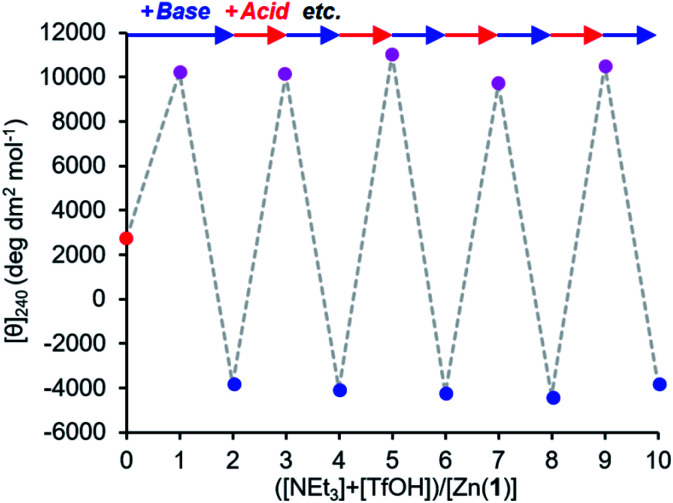
Molar ellipticity at 240 nm of Zn(1)·2ClO_4_ (*c*_0_ = 0.25 mM) with 2-H (1 eq.) and 4-H (1 eq.) in MeCN with alternating addition of NEt_3_ (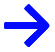) and TfOH (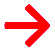) in 1 eq. increments.

The reversibility of the ligand exchange was explored using a base/acid cycling experiment. 2 equiv. of triethylamine was first added, with the first equiv. inducing binding of the phosphate ([*θ*]_240_ ∼ +10 000 deg dm^2^ mol^−1^), and the second inducing binding of the carboxylate ([*θ*]_240_ ∼ −4000 deg dm^2^ mol^−1^) (Fig. 7). Next, 1 equiv. of TfOH was added, causing protonation of the more basic carboxylate ligand and allowing the phosphate to rebind to the metal centre and give its characteristic CD output of +10 000 deg dm^2^ mol^−1^. The system was returned to the carboxylate-bound state by addition of another equivalent of NEt_3_ to deprotonate 2-H again. In this way, the system was reversibly switched between phosphate- and carboxylate-bound states over 4 complete cycles of alternate NEt_3_/TfOH addition, with minimal change in output magnitude for each state.

**Fig. 8:**
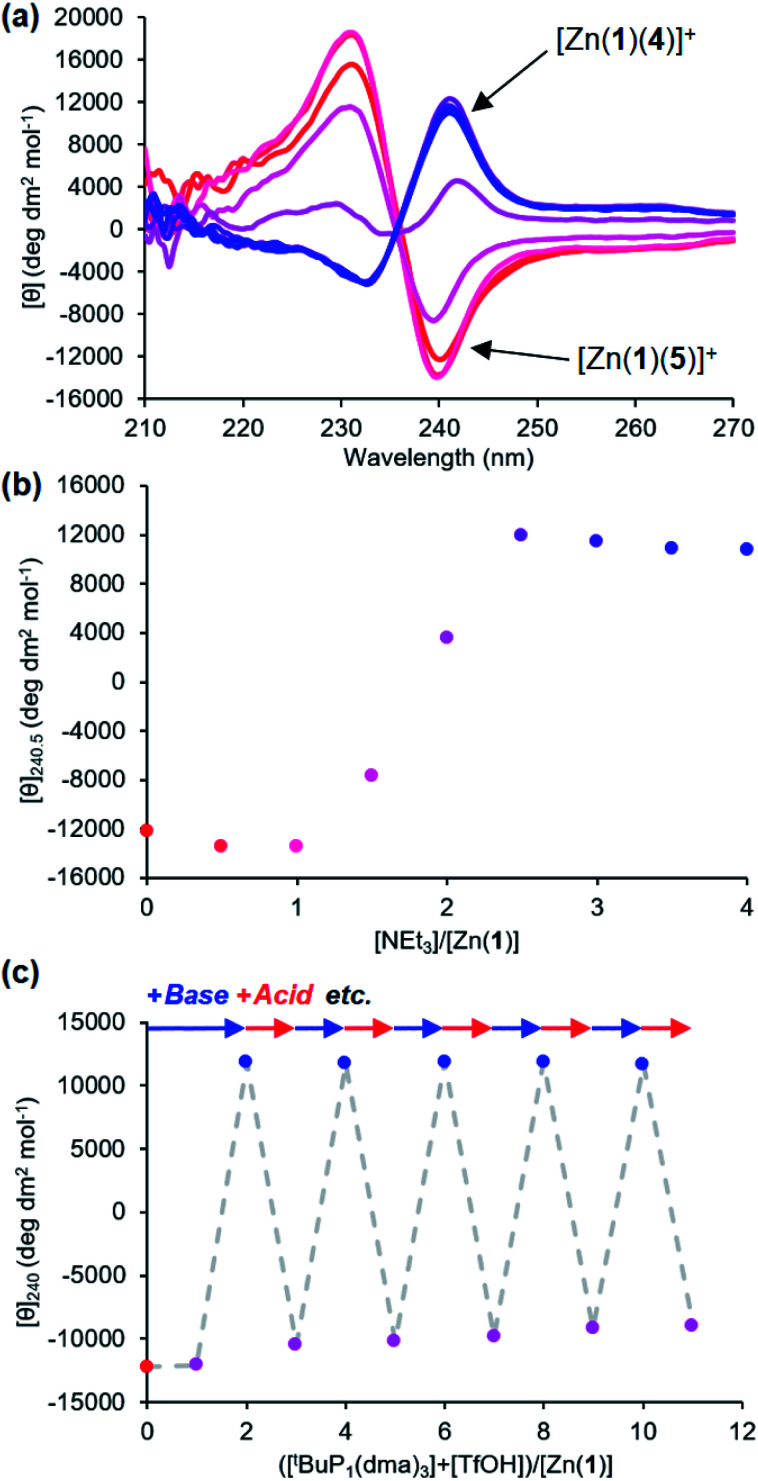
(a) CD spectra of Zn(1)·2ClO_4_ (*c*_0_ = 0.25 mM) with 4-H (1 eq.) and 5-H (1 eq.) in MeCN with addition of NEt_3_ (
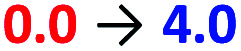
 eq.) in 0.5 eq. increments; (b) molar ellipticity at 240 nm with addition of NEt_3_; (c) molar ellipticity at 240 nm with alternating addition of ^t^BuP_1_(dma)_3_ (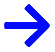) and TfOH (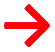) in 1 eq. increments.

It was also possible to exchange 4^−^ and 5^−^ at the metal centre by modulating pH. A solution 0.25 mM equimolar in Zn(1)·2ClO_4_ and in the two ligands was titrated with triethylamine. Initial binding of 5^−^ was indicated by a negative CD signal that swung to positive as a second equivalent of base deprotonated 4-H and induced ligand exchange (Fig. 8). Like the phosphate/carboxylate system, more than two equiv. of base were required to reach the maximum signal for the less acidic ligand. In contrast, the signal for the phosphate-bound state in this two-ligand system was close in magnitude as in the single ligand system. The negative charge on the phosphoramide ligand is sufficiently dispersed to not require as much anion stabilisation as the phosphate, reducing the favourability of forming doubly bound Zn(1) species. Base/acid cycling using the phosphazene base ^t^BuP_1_(dma)_3_ (p*K*_aH_(MeCN) = 26.98) and TfOH gave clean switching between phosphoramide-bound and phosphate-bound states over 5 complete base/acid cycles.

**Fig. 9:**
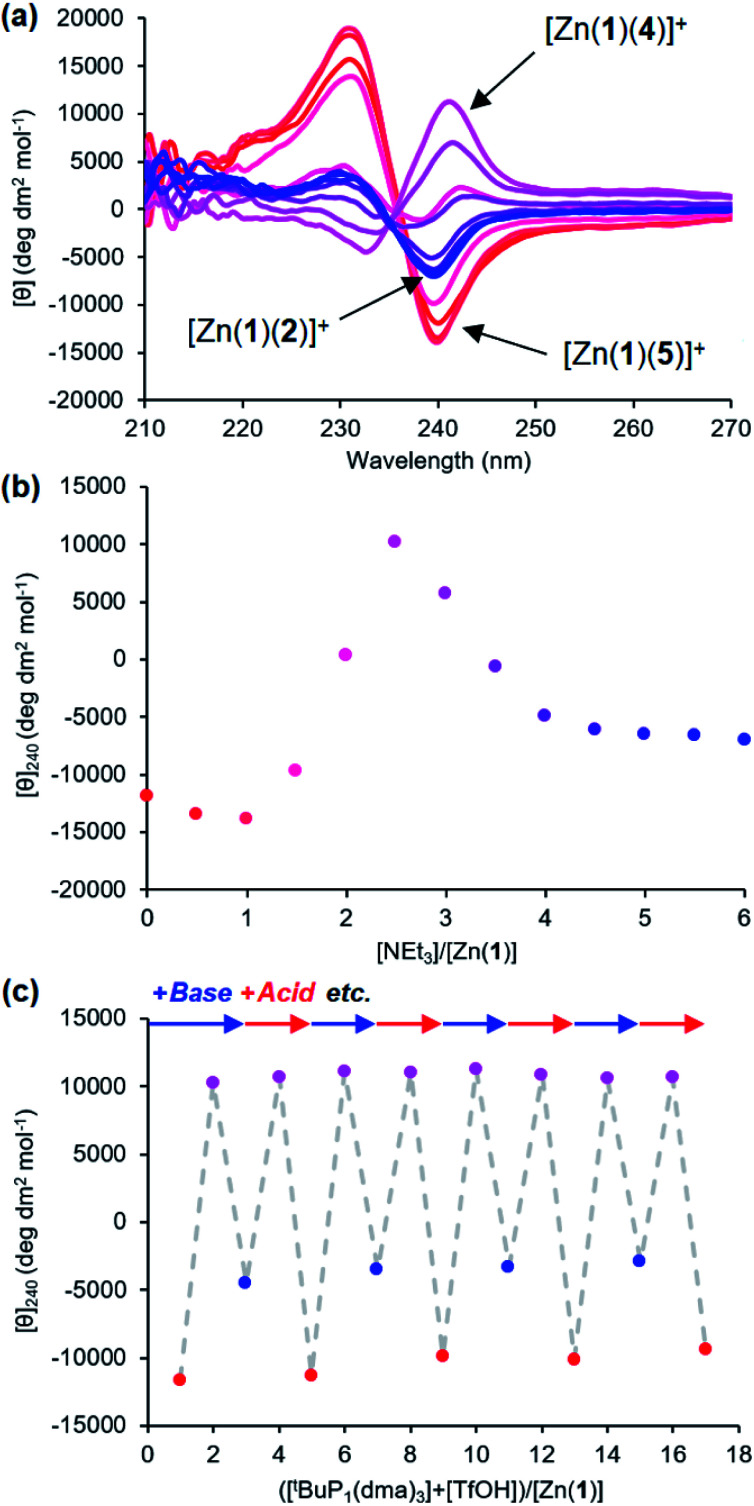
(a) CD spectra of Zn(1)·2ClO_4_ (*c*_0_ = 0.25 mM) with 2-H (1 eq.), 4-H (1 eq.) and 5-H (1 eq.) in MeCN with addition of NEt_3_ (
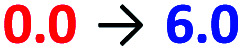
 eq.) in 0.5 eq. increments; (b) molar ellipticity at 240 nm with addition of NEt_3_; (c) molar ellipticity at 240 nm with alternating addition of ^t^BuP_1_(dma)_3_ (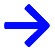) and TfOH (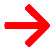) in 1 eq. increments.

Provided ligands can be found with sufficiently differentiated basicities, and hence binding constants, selective exchange among ligands in multicomponent mixtures should be possible, allowing fine-tuning of a conformational response to the environment of a complex chemical system. We created such a system by preparing a 0.25 mM solution of the four components Zn(1)·2ClO_4_, 2-H, 4-H and 5-H, and then by adding a fifth, triethylamine. Over the course of addition of 6 equiv. of triethylamine (Fig. 9a and b), the system responded as we had hoped with a series of ligand exchanges and consequent conformational responses. An initial negative CD response arising from the most acidic ligand 5^−^ persisted until more than 1 equiv. NEt_3_ was added, when the signal swung to a positive maximum at 2.5 equiv. of NEt_3_ due to deprotonation and binding of 4^−^. Further addition of NEt_3_ caused the signal to return to a negative value as the least acidic ligand 2^−^ was deprotonated, displacing 4^−^ from the metal centre. The number of equivalents of base required to deprotonate each ligand were consistent with the respective two-acid system *i.e.* 1.5 equivalents of NEt_3_ were required to deprotonate 4-H in the phosphoramide/phosphate system, while 3 equivalents of NEt_3_ were required to deprotonate 2-H in the phosphate/carboxylate magnitude and line shape, with the initial phosphoramide signal having a significantly larger negative lobe at 230 nm than the carboxylate signal. Two isosbestic points were observed at ∼235 and ∼236 nm, which is consistent with switching between just three different states.

This three-ligand system was successfully switched between each state using ^t^BuP_1_(dma)_3_/TfOH addition. First, 3 eq. of ^t^BuP_1_(dma)_3_ was added in 1 eq. increments to the same starting mixture as above, which gave the expected negative–positive–negative CD output pattern as the Zn(1) binding site passed through the phosphoramide-, phosphate- and carboxylate-bound states (Fig. 9c). Next, 2 equiv. of TfOH was added in 1 equiv. increments; the first equivalent protonated 2^−^, allowing 4^−^ to bind ([*θ*]_240_ ∼ +10 700 deg dm^2^ mol^−1^), while the second equivalent protonated 4^−^, returning the system to the 5^−^-bound state ([*θ*]_240_ ∼ −11 300 deg dm^2^ mol^−1^). With further alternating addition of 2 eq. of ^t^BuP_1_(dma)_3_ and TfOH in 1 eq. increments, the system was successfully shuttled between the three states for three additional cycles, though with a small amount of signal diminution for the phosphoramide- and carboxylate-bound states.

### Communicating the conformational response to a spatially remote site

Having demonstrated that pH-induced exchange of metal-bound ligands could be used as a mechanism for the conformational communication of information, we next used it to induce a more extensive conformational change by relaying the induced conformation of the BQPA ligand into an induced screw-sense preference in a pendent foldamer. Complex Zn(8)·2ClO_4_ was synthesised (see the ESI†), in which the achiral helical tetramer of the quaternary amino acid Aib^48^ was ligated at its N-terminus to the Zn(1) binding site, and at its C terminus to a fifth (*R*)-Aib* residue that was enantioselectively labelled with ^13^C in its pro-*R* enantiotopic methyl groups.^49^ Peak separation in the ^13^C-NMR spectrum allows such Aib* residues to act as reporters of local conformation,^50^ thus indicating that pH has been translated into a conformational change and relayed to a remote site. Attaching the foldamer at the pyridine 3-position was crucial to the function of the system; no ligand binding was observed with a 2-CO_2_Me substituted Zn(1) derivative, presumably due to increased steric congestion at the metal centre.

**Fig. 10:**
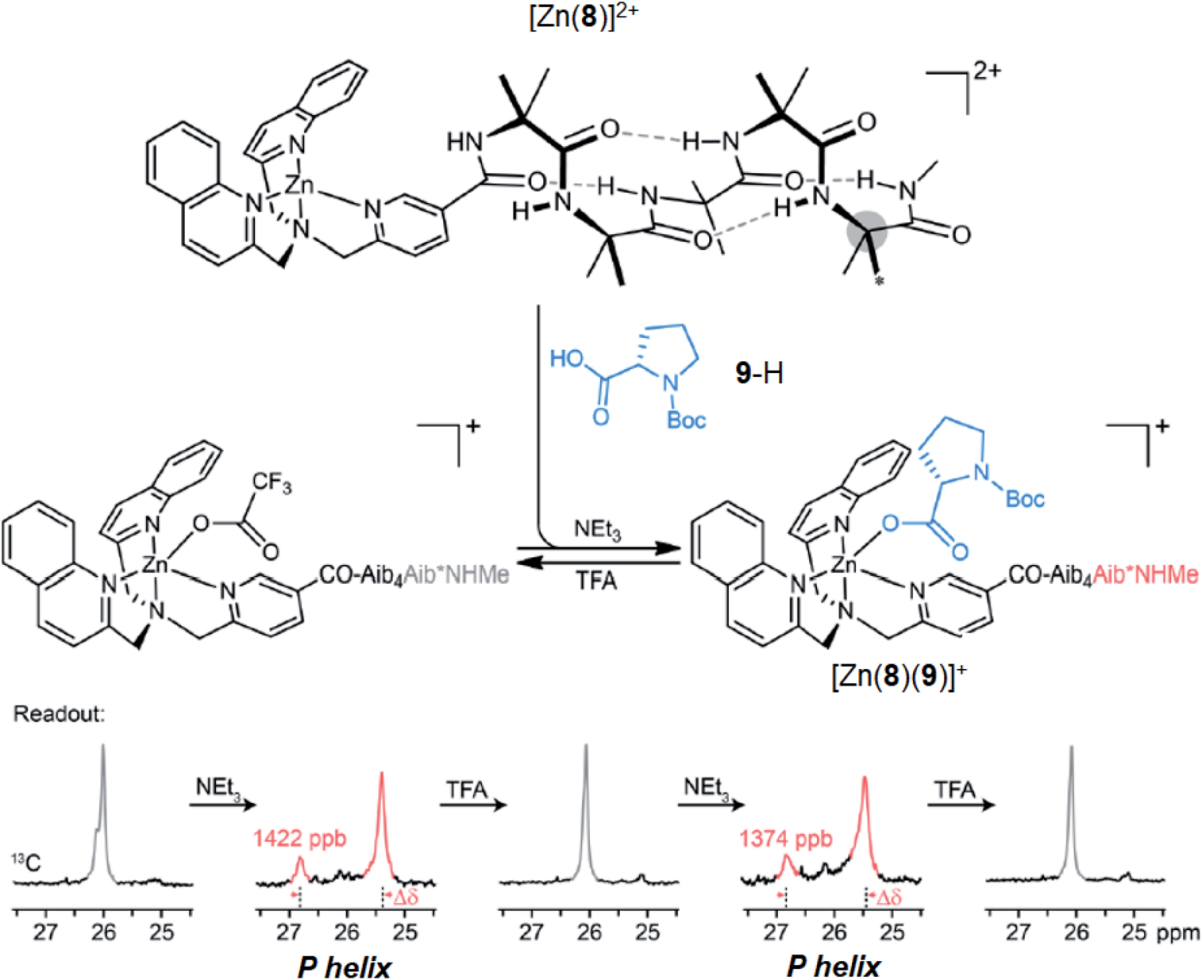
pH-induced binding of 9-H with complex [Zn(8)]^2+^ (*c*_o_ = 12.1 mM) with alternating addition of NEt_3_ and TFA in 1.2 eq. increments, monitored by ^13^C NMR of the terminal Aib*NHMe reporter.

A solution of Zn(8)·2ClO_4_ (*c*_o_ = 12.1 mM) and Boc-l-Pro-OH 9-H (1.2 equiv.) was prepared in MeCN-d_3_. In the absence of base, a very small separation (0.1 ppm) between the labelled methyl groups of Aib*NHMe was observed by ^13^C-NMR, indicating that the helical foldamer adopted both screw senses in essentially equal proportions (Fig. 10). On addition of a small excess of triethylamine (1.26 eq.), the characteristic major and minor labelled signals of the activated complex [Zn(8)(9)]^+^ appeared, separated by *ca.* 1.5 ppm, with the major signal upfield of the minor, indicating that a *P* helix^51^ was preferentially induced. Addition of a further excess of trifluoroacetic acid (TFA, 1.32 equiv.) reprotonated the proline, preventing its interaction with the binding site and once more giving a singlet in the ^13^C-NMR spectrum. Because the TFA itself now deprotonated, it is likely that it is complexed to the Zn centre. The signalling pathway was then switched on and off again, increasing the excess of triethylamine/TFA added each time by approximately 5% with respect to Zn(8)^2+^. There was almost no difference in the separation (anisochronicity) of the ^13^C signals (0.048 ppm) after successive additions of base.

**Fig. 11:**
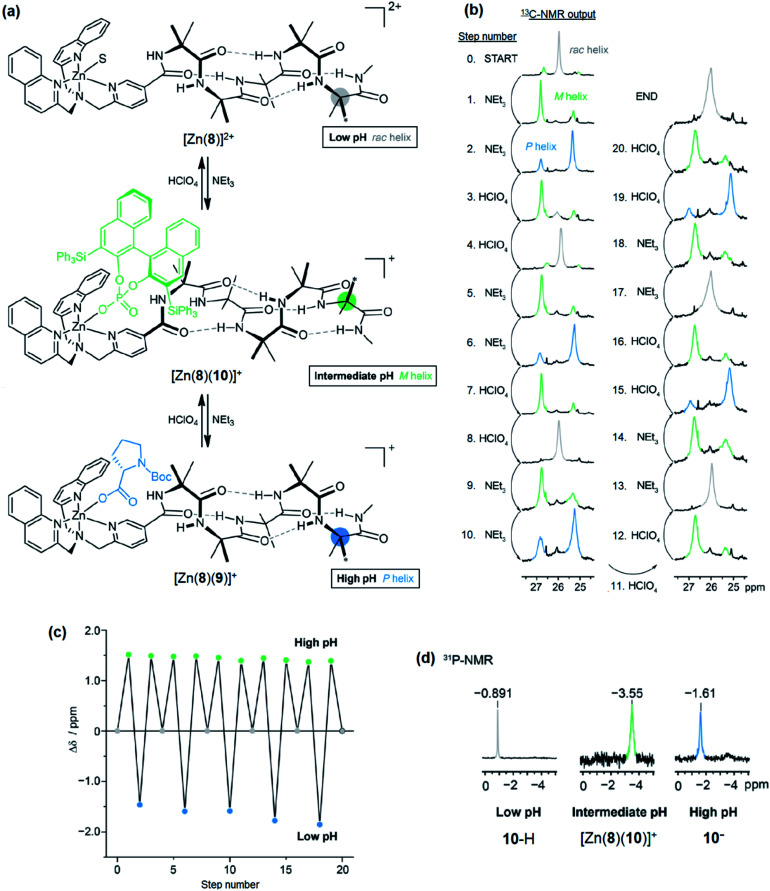
(a) pH-induced exchange of 9^−^ and 10^−^ with [Zn(8)]^+^, (b) ^13^C-NMR output from the terminal ^13^C-labelled Aib reporter group, (c) difference in chemical shift between major and minor peaks (*δ*_major_ − *δ*_minor_) after addition, (d) ^31^P NMR spectrum of the system at low, intermediate and high pH.

Next, a relay system capable of switching between *M*, *P* and off states based on pH was set up (Fig. 11a). A solution (*c*_o_ = 12.5 mM) of Zn(8)·2ClO_4_, 9-H (1.1 eq.) and (*S*)-3,3′-bis(triphenylsilyl)-1,1′-binaphthyl-2,2′-diyl hydrogenphosphate (10-H, 1.1 eq.) was prepared in MeCN-d_3_. As a bulky *C*_2_-symmetric phosphate, the binding behaviour of 10-H is similar to 4-H. We were no longer limited to CD-silent ligands for NMR studies and could optimise for screw sense preference induction. In the absence of base, the major species (85%) was the solvent-bound complex, indicated by the ^13^C-NMR singlet of the Aib*NHMe labelled methyl groups (Fig. 11b, step 0). A small amount of phosphate-bound complex [Zn(8)(10)]^+^ (15%) was also observed. Addition of NEt_3_ (1.1 equiv., step 1) formed [Zn(8)(10)]^+^ quantitatively and characteristic separation of the ^13^C-labelled methyl group signals was observed in ^13^C NMR spectrum. The major peak was downfield of the minor peak, indicating the formation of an *M* helix. A second addition of NEt_3_ (2.2 eq. total, step 2) reversed the positions of the major and minor labels in the NMR spectrum, indicating 9-H had been deprotonated and displaced 10^−^ to form [Zn(8)(9)]^+^ and induced a *P* helical preference. Addition of HClO_4_ (1.1 equiv, step 3) reprotonated 9^−^, leaving 10^−^ to bind to Zn and the conformational preference reverted to an *M* helix. A second addition of HClO_4_ (2.2 equiv. total, step 4) protonated 10^−^ as well, leaving the solvent-bound complex in the off state with no helical preference. Subsequent additions of NEt_3_ and HClO_4_ (steps 5–20) reversibly switched the conformational preference between all three states through five complete cycles (Fig. 11c), although it was necessary to increase the number of equivalents of NEt_3_/HClO_4_ for later additions to ensure that the system fully switched to the next state. This was attributed to the build-up of mildly acidic triethylammonium cations in the system, which may interfere with the acid–base equilibria of the ligands, particularly of the most basic carboxylate ligand 9^−^. From beginning to end, the chemical shift difference between the major and minor labels of Aib*NHMe in each state stayed almost constant, varying by at most 10%, illustrating the complete reversibility of this signalling pathway in communicating the stereochemical information of the ligand through conformational preference of the foldamer chain.

The binding of 10^−^ was also analysed by ^31^P-NMR (Fig. 11d). At all pH values, a single peak was observed in the ^31^P NMR spectrum. At low pH, 10-H is the major species, indicated by the chemical shift of *δ* −0.891 ppm. On deprotonation (intermediate pH), [Zn(8)(10)]^+^ is formed, accompanied by an upfield shift to *δ* −3.55 ppm. At high pH, free 10^−^ is formed due to displacement by 9^−^, leading to a downfield shift to *δ* −1.61 ppm.

## Conclusions

We have demonstrated pH-controlled ligand exchange at a host metal complex [Zn(1)]^2+^ with a set of three chiral ligands (2-H, 4-H and 5-H). The large separation between the p*K*_a_ values of the ligands allowed them to be independently protonated/deprotonated by stoichiometric quantities of acid and base. The differences between the association constants of the ligands were also sufficiently large that only one equivalent of each ligand (with respect to the host) was required for the system to function. By exploiting the chiroptical properties of [Zn(1)]^2+^, the identity of the bound ligand could be converted into a circular dichroism spectroscopy output; the chirality of the ligands were tuned such that upon binding to the host complex, each ligand induces a Cotton effect of opposite sign to the ligand(s) preceding or following it in the acidity sequence. The system was fully reversible, with minimal loss of signal magnitude after four base/acid addition cycles.

Exchange between two ligands was coupled to a conformational response in foldamer-appended complex [Zn(8)]^2+^. The foldamer preferentially adopted either an *M* or *P* conformation, depending on the identity of the bound ligand, which was then translated into an NMR output by a remote ^13^C-labelled Aib unit. This system was also fully reversible over five cycles of base/acid addition, with little variation in output magnitude.

Powerful pH homeostasis in natural systems means that exploiting p*K*_a_ differences to control the protonation state of a set of competitive ligands *in situ* is a process that nature is unable to exploit. However, the selective activation of a ligand by deprotonation above a given pH is reminiscent of the use of choline as a ‘proligand’ for the acetylcholine receptor, to which it binds only in its reversibly acetylated state.^52,53^ The use of a metal cation to mediate binding parallels the function of metal cofactors in metalloproteins, and the established ability of Zn^2+^-chiral anion interactions to persist in water suggests that a modified version of this chemical information processing system based on pH-gated binding might in future be used in aqueous systems.

## Data availability

Synthetic procedures, characterisation of novel compounds, CD/NMR titration methodology, miscellaneous CD spectra and p*K*_a_ calculation methodology may be found in the ESI.†

## Author contributions

J. C. conceived and supervised the study. M. M. W. and B. A. F. L. carried out the synthesis. M. M. W. performed the CD studies. B. A. F. L. performed the NMR studies. S. T. and I. L. performed the computational studies. All authors contributed to writing the manuscript.

## Conflicts of interest

There are no conflicts to declare.

## Supplementary Material

SC-013-D1SC06812A-s001
