# Supplementary material for: Inducing a pH-dependent conformational response by competitive binding to Zn2+ of a series of chiral ligands of disparate basicity
Source: Chem Sci. 2022 Jan 17;13(8):2258–69. doi: 10.1039/d1sc06812a (PMC8864710; doi:10.1039/d1sc06812a)
Supplement: SC-013-D1SC06812A-s001 [file SC-013-D1SC06812A-s001.pdf]

Electronic Supplementary Material (ESI) for Chemical Science.
This journal is © The Royal Society of Chemistry 2022

Contents

S1. General information & Instrumentation

S2. Synthetic details

S3. CD spectroscopy methodology

S4. Miscellaneous CD spectra

S5. NMR titration methodology

S6. pK_a calculation methodology

S7. References

S1. General information & Instrumentation

Air- and/or moisture sensitive reactions were performed under a nitrogen atmosphere with glassware that was oven-dried or dried by heat gun, using standard anhydrous techniques. Air- and/or moisture sensitive liquids and solutions were transferred via syringe through rubber septa.

All reagents were purchased from commercial suppliers and used as received, unless otherwise stated. Triethylamine was stored over potassium hydroxide pellets. Anhydrous dichloromethane (DCM) and tetrahydrofuran (THF) were dried using an Anhydrous Engineering Grubbs-type solvent system, or by distillation over calcium hydride (DCM) or sodium wire (THF). Anhydrous acetonitrile (MeCN) was purchased from Acros Organics and was measured to have a water content of 7.1 ppm by Karl Fischer titration (average of 3 measurements). Non-anhydrous solvents were purchased from commercial suppliers and used as received. P.E. refers to fractions of petroleum ether with boiling points in the range 40–60 ^{o}C.

Flash column chromatography was performed on silica gel (VWR 40-63 µm, or Merck 60H 40-60 µm, 230-300 mesh) or alumina (Fisher, Brockmann grade 1, neutral). Analytical thin-layer chromatography (TLC) was performed on aluminium-backed silica (Merck 60 Å F_{254}) or alumina (Aldrich 60Å F_{254}) plates, visualising using a UV lamp (254 nm) or potassium permanganate stain.

^{1}H, ^{13}C and ^{31}P NMR spectra were acquired on Bruker (300, 400, or 500 MHz) or Jeol ECS (400 MHz) spectrometers. Chemical shifts (δ) are reported in parts per million (ppm) and coupling constants (*J*) are reported in Hertz (Hz) to the nearest 0.1 Hz. ^{1}H-NMR spectra are referenced to the residual solvent peak ($CDCl_3$ 7.26, CD_3OD 3.31, D_2O 4.79, DMSO-d_6 2.50) and ^{13}C-NMR were referenced to the ^{13}C resonance of the solvent ($CDCl_3$ 77.16, CD_3OD 49.00, DMSO-d_6 39.52) except in the case of D_2O where MeOH was used as an internal standard and was set to 49.5 ppm. Multiplicities are reported as singlet (s), doublet (d), triplet (t), quartet (q), multiplet (m), broad (br) or combinations thereof. Major and minor rotamers/diastereomers are denoted by the subscripts 'maj' and 'min'.

Low- and high-resolution mass spectra were recorded by staff at the Universities of Bristol and Manchester. High-resolution ESI spectra were acquired on Bruker micrOTOF II, Thermo Scientific Orbitrap Elite or Thermo Scientific Finnigan MAT95XP spectrometers. Low-resolution ESI spectra were acquired on a Waters Platform II spectrometer. High-resolution nanospray spectra were acquired on a Waters Synapt G2S spectrometer with Advion Nanomate installed.

Infrared spectra were recorded on Perkin Elmer Spectrum Two or Spectrum RX1 FT-IR spectrometers. Only absorption maxima (ν_{max}) of interest above 1000 cm^{-1} are reported and quoted in wavenumbers (cm^{-1}).

CD spectra were acquired on a JASCO J-815 CD spectrometer at 25 ^{o}C using a 1 mm path length quartz cuvette. See section S**3** for detailed descriptions of sample preparation and experimental methods.

S2. Synthetic details

N,*N*-bis(quinolin-2-ylmethyl)-*N*-(pyridin-2-ylmethyl)amine (BQPA) **1**

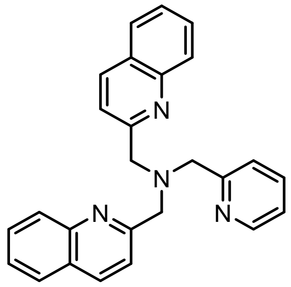

2-Aminomethylpyridine (0.11 ml, 1.0 mmol) was added to a solution of 2-bromomethylquinoline (444 mg, 2.0 mmol) and DIPEA (0.52 mL, 3.0 mmol) in THF (7.5 ml) and the resulting mixture stirred at room temperature for 3 days. The reaction mixture was filtered and concentrated. The residue was purified by alumina column chromatography (3:1 EtOAc:P.E., R_f = 0.2) to give the title compound (297 mg, 76%) as a yellow solid.

^{1}H NMR (400 MHz, CDCl$_3$): δ_H 8.55–8.53 (m, 1H, PyH), 8.12 (d, *J* = 8.5, 2H, QuinH), 8.05 (d, *J* = 8.5, 2H, QuinH), 7.78–7.74 (m, 4H, QuinH), 7.69–7.63 (m, 3H, QuinH + PyH), 7.59 (dt, *J* = 7.8, 1.3, 1H, PyH), 7.49 (ddd, *J* = 8.1, 6.9, 1.2, 2H, QuinH), 7.15–7.11 (m, 1H, PyH), 4.09 (s, 4H, CH$_2$-Quin), 3.97 (s, 2H, CH$_2$-Py)

^{13}C NMR (101 MHz, CDCl$_3$): δ_C 160.3 (C_{Quin}), 159.4 (C_{Py}), 149.3 (C_{Py}H), 147.8 (C_{Quin}), 136.5 (C_{Py}H + C_{Quin}H), 129.5 (C_{Quin}H), 129.3 (C_{Quin}H), 127.6 (C_{Quin}H), 127.5 (C_{Quin}), 126.3 (C_{Quin}H), 123.4 (C_{Py}H), 122.2 (C_{Py}H), 121.2 (C_{Qu}H), 61.2 (CH$_2$), 60.6 (CH$_2$)

Spectroscopic data are consistent with those previously reported.[1]

Zn(**1**).2ClO$_4$

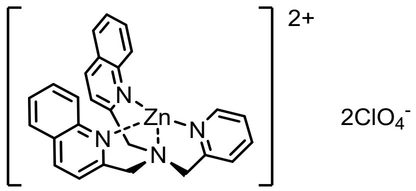

BQPA **1** (195 mg, 0.5 mmol) was dissolved in MeOH (10 mL), then Zn(ClO$_4$)$_2$.6H$_2$O (186 mg, 0.5 mmol) was added dropwise as a solution in MeOH (5 mL). The mixture was stirred at room temperature for 10 minutes, then Et$_2$O (45 mL) was added. After stirring for 1 hour at room temperature, the solvent was decanted from the yellow residue that had formed. The residue was triturated with Et$_2$O, then dried under vacuum to give the title compound (196 mg, 60%) as a yellow solid.

^{1}H NMR (400 MHz, CD$_3$OD): δ_H 8.78 (d, *J* = 5.3, 1H, PyH), 8.55 (d, *J* = 8.7, 2H, QuinH), 8.50 (d, *J* = 8.5, 2H, QuinH), 8.00–7.92 (m, 5H, PyH + QuinH x2), 7.68 (t, *J* = 7.6, 2H, QuinH), 7.61–7.56 (m, 3H, PyH + QuinH), 7.50 (t, *J* = 6.6, 1H, PyH), 4.79 (d, *J* = 17.3, 2H, C**H**H-Quin), 4.76 (d, *J* = 17.3, 2H, CH**H**-Quin), 4.60 (s, 2H, C**H**$_2$-Py)

^{13}C NMR (101 MHz, CD_3OD): δ_C 159.0, 155.9, 148.5, 146.2, 142.5, 142.2, 133.1, 130.1, 130.0, 129.1, 126.7, 126.3, 126.0, 122.6, 62.6, 61.3

IR: 3303 (br), 3081, 3029, 2924, 1604, 1515, 1434, 1090

HRMS (Nanospray$^+$, MeOH): Calc for $C_{26}H_{22}N_4{}^{64}Zn$ ($[M]^{2+}$) 227.0568, found 227.0574

Boc-D-Pro-OMe **2**-Me

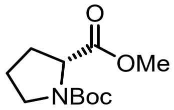

Methyl iodide (50 µL, 0.80 mmol) and potassium carbonate (207 mg, 0.75 mmol) were added to a solution of Boc-D-proline (108 mg, 0.5 mmol) in DMF (5 mL). The mixture was stirred at room temperature for 16 h, then quenched by the addition of sat. ammonium chloride. The mixture was extracted with diethyl ether, and the combined organic layers were washed with brine, dried (Na_2SO_4) and concentrated to give the title compound (95 mg, 83%) as a pale yellow oil. Isolated as a 40:60 mixture of rotamers.

^{1}H NMR ($CDCl_3$, 400 MHz): δ 4.28 (dd, *J* = 8.6, 3.3, 0.4H, $C^{\alpha}H_{min}$), 4.18 (dd, *J* = 8.5, 4.2, 0.6H, $C^{\alpha}H_{maj}$), 3.68 (s, 3H, OCH_3), 3.54–3.30 (m, 2H, $C^{\delta}H_2$), 2.24–2.08 (m, 1H, C^{β}**H**H), 1.96–1.77 (m, 3H, $C^{\gamma}H_2$ + C^{β}H**H**), 1.42 (s, 3.4H, $C(CH_3)_{3,min}$), 1.37 (s, 5.6H, $C(CH_3)_{3,maj}$)

^{13}C NMR ($CDCl_3$, 101 MHz): δ 173.8 (C**C**=$O_{,maj}$), 173.6 (C**C**=$O_{,min}$), 154.5 (N**C**=$O_{,min}$), 153.8 (N**C**=$O_{,maj}$), 79.9 (**C**$(CH_3)_{3,maj}$), 79.8 (**C**$(CH_3)_{3,min}$), 59.2 ($C^{\alpha}H_{,maj}$), 58.8 ($C^{\alpha}H_{,min}$), 52.1 ($OCH_{3,min}$), 52.0 ($OCH_{3,maj}$), 46.6 ($C^{\delta}H_{2,min}$), 46.4 ($C^{\delta}H_{2,maj}$), 30.9 ($C^{\beta}H_{2,maj}$), 30.0 ($C^{\beta}H_{2,min}$), 28.5 (C(**C**$H_3)_{3,min}$), 28.4 (C(**C**$H_3)_{3,maj}$), 24.4 ($C^{\gamma}H_{2,min}$), 23.7 ($C^{\gamma}H_{2,maj}$)

Spectroscopic data are consistent with those previously reported for Boc-L-Pro-OMe.[2]

Tetrabutylammonium *tert*-butyl-D-prolinate **2**.TBA

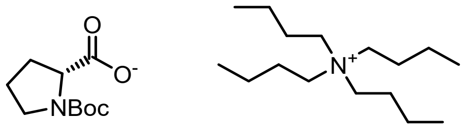

Tetrabutylammonium hydroxide (1.0 mL, 1 M in methanol, 1.0 mmol) was added to a solution of Boc-D-Pro-OH (215 mg, 1.0 mmol) in methanol (10 mL), The mixture was stirred at room temperature for 1 hour, then the solvent was evaporated. Azeotropic removal of water with toluene followed by trituration of the residue with hexane gave the desired salt (447 mg, 98%) as a colourless hygroscopic solid, which was stored in a vacuum desiccator over $CaCl_2$. Isolated as a 2:1 mixture of rotamers

^{1}H NMR (400 MHz, $CDCl_3$): δ_H 4.07 (dd, *J* = 7.7, 4.1, 0.33H, $C^{\alpha}H_{min}$), 3.99 (dd, *J* = 7.5, 2.8, 0.67H, $C^{\alpha}H_{maj}$), 3.57–3.47 (m, 1H, C^{δ}**H**H), 3.40–3.19 (m, 9H, C^{δ}H**H** + $N(CH_2)_4$), 2.10–1.80 (m, 3H, $C^{\beta}H_2$ + C^{γ}**H**H), 1.69–1.52 (m, 9H, C^{γ}H**H** + CH_2 x4), 1.45–1.31 (m, 17H, $C(CH_3)_3$ + CH_2 x4), 0.93 (t, *J* = 7.3, 12H, CH_3 x4)

^{13}C NMR (101 MHz, CDCl$_3$): δ_C 176.8 (C**C**=O), 155.3 (NC=O$_{min}$), 154.6 (NC=O$_{maj}$), 78.0 (**C**(CH$_3$)$_{3,min}$), 77.4 (**C**(CH$_3$)$_{3,maj}$), 62.4 (C$^{\alpha}$H), 58.5 (NCH$_2$), 46.8 (C$^{\delta}$H$_{2,maj}$), 46.4 (C$^{\delta}$H$_{2,min}$), 31.9 (C$^{\beta}$H$_{2,min}$), 31.2 (C$^{\beta}$H$_{2,maj}$), 28.7 (C(**C**H$_3$)$_3$), 24.4 (C$^{\gamma}$H$_{2,maj}$), 24.2 (CH$_2$), 23.7 (C$^{\gamma}$H$_{2,min}$), 19.8 (CH$_2$), 13.8 (CH$_3$)

IR: 2963, 2876, 1678, 1590, 1410, 1387, 1165

HRMS (Nanospray$^-$, CHCl$_3$): Calc. for $C_{10}H_{16}NO_4$ ([M]$^-$) 214.1079, found 214.1074

3-(((3aR,8aR)-2,2-dimethyl-6-oxido-4,4,8,8-tetraphenyltetrahydro-[1,3]dioxolo[4,5-e][1,3,2] dioxaphosphepin-6-yl)oxy)propanenitrile **4**-CH$_2$CH$_2$CN

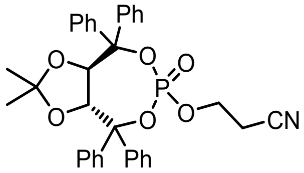

Prepared according to a modified literature procedure.[3] Phosphorus trichloride (138 µL, 1.58 mmol) was added dropwise to a solution of (4*R*,5*R*)-2,2-Dimethyl-α,α,α′,α′-tetraphenyldioxolane-4,5-dimethanol (700 mg, 1.5 mmol) and NEt$_3$ (0.71 mL, 5.1 mmol) in THF (5 mL) at 0 °C. After stirring for 1 h at 0 °C, a solution of 3-hydroxypropionitrile (0.12 mL, 1.8 mmol) in THF (1 mL) was added dropwise at 0 °C. The mixture was stirred at room temperature for 2 h, then filtered and evaporated. The residue was redissolved in DCM (5 mL) and cooled to 0 °C. mCPBA (388 mg, 2.25 mmol) was added, and the mixture was stirred at room temperature for 40 min. then quenched by the addition of NaHCO$_3$. The organic layer was washed with brine, then dried (MgSO$_4$), filtered and concentrated. The crude product was purified over silica (Et$_2$O, R$_f$ = 0.25) to give the title compound (692 mg, 79%) as a colourless solid.

^{1}H NMR (400 MHz, CDCl$_3$): δ_H 7.60–7.52 (m, 4H, ArH), 7.46–7.27 (m, 16H, ArH), 5.43 (d, *J* = 8.1, 1H, CH), 5.15 (d, *J* = 8.1, 1H, CH), 3.94–3.87 (m, OC**H**H), 3.45–3.37 (m, 1H, OCH**H**), 2.25–2.17 (m, 1H, C**H**HCN), 2.02–1.94 (m, 1H, CH**H**CN), 0.85 (s, 3H, CH$_3$), 0.50 (s, 3H, CH$_3$)

^{13}C NMR (101 MHz, CDCl$_3$): δ_C 143.9 (d, *J* = 1.4, Ar), 143.3 (d, *J* = 4.4, Ar), 139.6 (d, *J* = 6.7), 139.1 (d, *J* = 10.0, Ar), 129.1 (Ar), 128.7 (Ar), 128.7 (Ar), 128.5 (Ar), 128.5 (Ar), 128.4 (Ar), 128.1 (Ar), 128.1 (Ar), 127.8 (Ar), 127.5 (Ar), 127.4 (Ar), 127.0 (Ar), 116.2 (C≡N), 114.0 (**C**(CH$_3$)$_2$), 89.0 (d, $^2J_{CP}$ = 7.5, **C**Ph$_2$), 88.5 (d, $^2J_{CP}$ = 8.2, **C**Ph$_2$), 80.1 (d, $^3J_{CP}$ = 1.3, CH), 78.3 (d, $^3J_{CP}$ = 0.7, CH), 62.1 (d, $2J_{CP}$ = 5.0, OCH$_2$), 27.0 (CH$_3$), 26.3 (CH$_3$), 19.1 (d, $^3J_{CP}$ = 8.6, **C**H$_2$CN)

Spectroscopic data are consistent with those previously reported.[3]

(3a*R*,8a*R*)-6-hydroxy-2,2-dimethyl-4,4,8,8-tetraphenyltetrahydro-[1,3]dioxolo[4,5-e][1,3,2] dioxaphosphepine 6-oxide ((−)-TADDOL-PO$_2$H) **4**-H

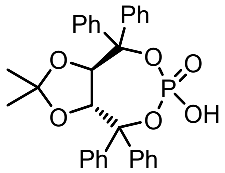

Prepared according to a modified literature procedure.[3] DBU (0.89 mL, 5.9 mmol) was added to a solution of **mw-331** (692 mg, 1.19 mmol) in DCM (10 mL), and the mixture was stirred at room temperature for 2.5 h. AcOH (0.5 mL) and water (5 mL) were added, and the organic layer was washed with HCl (0.5 M, aq.) and brine, then dried ($MgSO_4$), filtered and concentrated. The crude product was recrystallised from EtOAc/hexane to give the title compound (477 mg, 76%) as a colourless solid.

^{1}H NMR (400 MHz, $CDCl_3$): δ_H 8.79 (s, 1H, OH), 7.56–7.52 (m, 4H, ArH), 7.38–7.35 (m, 4H, ArH), 7.34–7.27 (m, 6H, ArH), 7.27–7.22 (m, 6H, ArH), 5.20 (s, 2H, CH x2), 0.64 (s, 6H, CH_3 x2)

^{13}C NMR (101 MHz, $CDCl_3$): δ_C 143.5 (d, J = 2.1, Ar), 139.7 (d, J = 8.8, Ar), 128.9 (Ar), 128.3 (Ar), 128.3 (Ar), 127.8 (Ar), 127.3 (Ar), 127.0 (Ar), 113.8 (**C**(CH_3)$_2$), 88.1 (d, J = 6.1, **C**Ph_2), 79.6 (CH), 26.7 (CH_3)

HRMS (Nanospray$^-$, DCM): Calc. for $C_{31}H_{28}O_6P$ ([M-H]$^-$) 527.1624, found 527.1638

Spectroscopic data are consistent with those previously reported.[3]

Tetrabutylammonium (3a*R*,8a*R*)-2,2-dimethyl-4,4,8,8-tetraphenyltetrahydro-[1,3]dioxolo[4,5-e][1,3,2]dioxaphosphepin-6-olate 6-oxide **4**.TBA

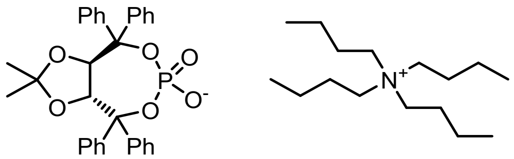

Tetrabutylammonium hydroxide (0.2 mL, 1 M in methanol, 0.2 mmol) was added to a suspension of **mw-332** (106 mg, 0.2 mmol) in methanol (2 mL), The mixture was stirred at room temperature for 1 hour, then the solvent was evaporated. Azeotropic removal of water with toluene followed by trituration of the residue with pentane gave the desired salt (142 mg, 92%) as a colourless solid. An analytically pure sample (50 mg, 32%) was obtained by recrystallising twice from EtOAc/hexane.

^{1}H NMR (400 MHz, $CDCl_3$): δ_H 7.71–7.68 (m, 4H, ArH), 7.63–7.59 (m, 4H, ArH), 7.23–7.09 (m, 12H, ArH), 5.33 (s, 2H, OC**H**), 3.03–2.99 (m, 8H, NC**H**$_2$CH$_2$), 1.44–1.35 (m, 8H, NCH$_2$C**H**$_2$), 1.34–1.23 (m, 8H, C**H**$_2$CH$_3$), 0.93 (t, J = 7.2, 12H, CH$_2$C**H**$_3$), 0.76 (s, 6H, C(C**H**$_3$)$_2$)

^{13}C NMR (101 MHz, $CDCl_3$): δ_C 148.1 (d, $^3J_{C-P}$ = 5.3, Ar), 143.4 (d, $^3J_{C-P}$ = 6.0, Ar), 129.8 (Ar), 127.7 (Ar), 127.4 (Ar), 126.7 (Ar), 126.3(Ar), 110.3 (**C**(CH_3)$_2$), 81.7 (d, $^2J_{C-P}$ = 6.3, **C**(Ph)$_2$), 81.2 (d, $^3J_{C-P}$ = 1.3, O**C**H)), 58.5 (N**C**H$_2$CH$_2$), 26.7 (C(**C**H$_3$)$_2$), 24.1 (NCH$_2$**C**H$_2$), 19.8 (**C**H$_2$CH$_3$), 13.9 (CH$_2$**C**H$_3$)

IR: 3058, 2962, 2935, 2875, 1492, 1447, 1266, 1055

HRMS (Nanospray$^-$, $CHCl_3$): Calc. for $C_{31}H_{28}O_6P$ ([M]$^-$) 527.1624, found 527.1609

(4*S*,5*S*)-2,2-Dimethyl-α,α,α′,α′-tetraphenyldioxolane-4,5-dimethanol ((+)-TADDOL)

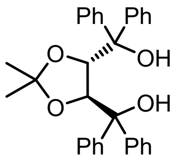

Prepared according to a modified literature procedure.[4] Phenylmagnesium bromide (3.37 mL, 2.6 M in hexane, 8.8 mmol) was dissolved in THF (6 mL) and cooled to 0 °C. (+)-Dimethyl 2,3-*O*-isopropylidene-D-tartrate (0.37 mL, 2.0 mmol) was added dropwise. The mixture was heated under reflux for 2 h, then cooled to room temperature and quenched with sat. NH_4Cl. THF was removed under reduced pressure and the residue was extracted with EtOAc. The combined organic extracts were washed with brine, then dried ($MgSO_4$) and concentrated. The crude residue was purified over silica (30:70 Et_2O:P.E., R_f = 0.23) to give the title compound (733 mg, 79%) as a colourless solid.

^{1}H NMR (400 MHz, $CDCl_3$): δ_H 7.54–7.51 (m, 4H, ArH), 7.36–7.21 (m, 16H, ArH), 4.60 (s, 2H, CH), 3.88 (s, 2H, OH), 1.04 (s, 6H, $C(CH_3)_2$)

^{13}C NMR (101 MHz, $CDCl_3$): δ_C 146.0 (Ar), 142.8 (Ar), 128.7 (Ar), 128.3 (Ar), 127.7 (Ar), 127.4 (Ar), 127.4 (Ar), 109.7 (**C**$(CH_3)_2$), 81.1 (CH), 78.3 (COH), 27.3 (C(**C**$H_3)_2$)

Spectroscopic data are consistent with those previously reported for the (–)- enantiomer.[5]

(3a*S*,8a*S*)-6-chloro-2,2-dimethyl-4,4,8,8-tetraphenyltetrahydro-[1,3]dioxolo[4,5-e][1,3,2] dioxaphosphepine 6-oxide ((+)-TADDOL-PO_2Cl)

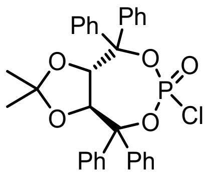

Prepared according to a modified literature procedure.[6] ^{n}BuLi (1.04 mL, 2.5 M in hexanes, 2.6 mmol) was added dropwise to a solution of (4S,5S)-2,2-Dimethyl-α,α,α′,α′-tetraphenyldioxolane-4,5-dimethanol (559 mg, 1.2 mmol) in THF (6 mL) at -78 °C. The mixture was warmed to room temperature and stirred for 1 h, then cooled to -78 °C. Phosphoryl chloride (0.15 mL, 1.6 mmol) was added dropwise, and the mixture was stirred for 1 h at -78 °C. After warming to room temperature, the mixture was stirred for 1 h then quenched by the addition sat. NH_4Cl (10 mL) and diluted with Et_2O (25 mL). The mixture was washed with sat. $NaHCO_3$ and brine, then dried ($MgSO_4$), filtered and concentrated. The crude product was recrystallised from EtOAc/hexane to give the title compound (357 mg, 54%) as a colourless solid.

^{1}H NMR (400 MHz, $CDCl_3$): δ_H 7.61–7.57 (m, 4H, ArH), 7.44–7.37 (m, 6H, ArH), 7.34–7.26 (m, 10H, ArH), 5.38 (d, J = 7.9, CH), 5.32 (d, J = 7.9, CH), 0.63 (s, 3H, CH_3), 0.57 (s, 3H, CH_3)

^{13}C NMR (101 MHz, $CDCl_3$): δ_C 142.2 (Ar), 141.5 (Ar), 138.9 (d, J = 10.4, Ar), 138.8 (d, J = 11.2, Ar), 129.1 (Ar), 129.0 (Ar), 128.9 (Ar), 128.6 (Ar), 128.5 (Ar), 128.4 (Ar), 128.3 (Ar), 128.2 (Ar), 127.6 (Ar), 127.6 (Ar), 127.5 (Ar), 127.0 (Ar), 115.2 (**C**$(CH_3)_2$), 92.7 (d, $^2J_{C\text{-}P}$ = 9.5, **C**$(Ph)_2$), 91.7 (d, $^2J_{C\text{-}P}$ = 10.9, **C**Ph_2), 79.6 (CH), 78.7 (CH), 26.7 (CH_3)

Spectroscopic data are consistent with those previously reported for the (–)- enantiomer.[6]

N-((3aS,8aS)-2,2-dimethyl-6-oxido-4,4,8,8-tetraphenyltetrahydro-[1,3]dioxolo[4,5-e][1,3,2]dioxaphosphepin-6-yl)-1,1,1-trifluoromethanesulfonamide (+)-TADDOL-PO_2NHTf **5**-H

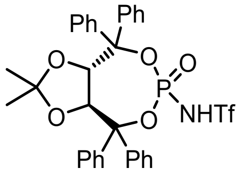

Prepared according to a modified literature procedure.[7] A mixture of **Mw-328** (302 mg, 0.55), trifluoromethanesulfonamide (165 mg, 1.1 mmol) and DIPEA (0.58 mL, 3.3 mmol) in chloroform (2 mL) was heated under reflux for 20 h. After evaporation of the solvent, the crude residue purified over silica (2:1 EtOAc:P.E., R_f = 0.37) to give the diisopropylethylammonium salt **5**.iPr$_2$EtNH. The salt was dissolved in EtOAc and washed with HBr (2 M aq.), and the organic layer dried (Na_2SO_4), filtered and concentrated. The residue was triturated with hexane and dried under vacuum to give the title compound (143 mg, 39%) as a pale-yellow solid.

^{1}H NMR (400 MHz, CDCl$_3$): δ_H 7.53–7.43 (m, 6H, ArH), 7.37–7.26 (m, 14H, ArH), 5.36 (d, *J* = 7.9, 1H, CH), 5.28 (d, *J* = 7.9, 1H, CH), 0.92 (s, 3H, CH_3), 0.44 (s, 3H, CH_3)

^{13}C NMR (126 MHz, CDCl$_3$): δ_C 143.7 (Ar), 143.1 (d, $^3J_{CP}$ = 6.8, Ar), 139.53 (Ar), 139.2 (d, $^3J_{CP}$ = 10.6, Ar), 129.1 (Ar), 128.5 (Ar), 128.3 (Ar), 128.1 (Ar), 127.6 (Ar), 127.5 (Ar), 127.1 (Ar), 119.5 (q, $^1J_{CF}$ = 323.2, CF_3), 113.6 (**C**(CH_3)$_2$), 90.4 (d, *J* = 5.8, **C**Ph$_2$), 88.8 (d, *J* = 3.3, **C**Ph$_2$), 80.1 (CH), 79.3 (CH), 27.3 (CH_3), 26.0 (CH_3)

IR: 3065, 2992, 2935, 1448, 1206, 1011

HRMS (ESI$^-$, MeCN): Calc. for $C_{32}H_{28}F_3NO_7PS$ ([M-H]$^-$) 658.1282, found 658.1253

<u>*tert*-Butyl (*S*)-2-carbamothioylpyrrolidine-1-carboxylate **6**-H</u>

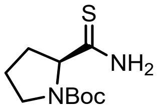

Boc-Pro-NH$_2$ (54 mg, 0.25 mmol) and Lawesson's reagent (55 mg, 0.14 mmol) were dissolved in THF (1 mL) and heated under reflux for 26 h. After cooling to room temperature, the solvent was evaporated and the crude residue purified over silica (5:95 MeOH:CHCl$_3$, R_f = 0.40) to give the title compound (59 mg, quant.) as a colourless solid. Isolated as a mixture of rotamers.

^{1}H NMR (400 MHz, DMSO-*d*$_6$): δ_H 9.52 (br s, 1H, NH), 9.12–9.07 (m, 1H, NH), 4.42–4.39 (m, 1H, C$^\alpha$H), 3.49–3.28 (m, partially obscured by H_2O peak, C$^\delta$H$_2$), 2.23–2.13 (m, 1H, C$^\beta$**H**H), 1.89–1.69 (m, 3H, C$^\beta$H**H** + C$^\gamma$H$_2$), 1.39–1.33 (m, 9H, C(CH_3)$_3$)

^{13}C NMR (101 MHz, DMSO-*d*$_6$): δ_C 209.0 (C=S$_{maj}$), 208.2 (C=S$_{min}$), 153.6 (C=O$_{min}$), 153.1 (C=O$_{maj}$), 78.5 (**C**(CH_3)$_3$), 66.4 (C$^\alpha$H), 47.0 (C$^\delta$H$_{2,min}$), 46.7 (C$^\delta$H$_{2,maj}$), 33.5 (C$^\beta$H$_{2,maj}$), 32.7 (C$^\beta$H$_{2,min}$), 28.1 (C(**C**H$_3$)$_{3,min}$), 28.0 (C(**C**H$_3$)$_{3,maj}$), 23.6 (C$^\gamma$H$_{2,min}$), 23.0 (C$^\gamma$H$_{2,maj}$)

HRMS (ESI$^+$, CHCl$_3$): Calc. for $C_{10}H_{18}N_2NaO_2S$ ([M+Na]$^+$) 253.0981, found 253.0981

Spectroscopic data are consistent with those previously reported.[8]

<u>Boc-L-Pro-NHOBn **7**-Bn</u>

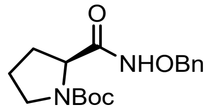

N-(3-Dimethylaminopropyl)-*N'*-ethylcarbodiimide hydrochloride (105 mg, 0.55 mmol) was added to a solution of Boc-L-Proline (108 mg, 0.5 mmol), *O*-benzylhydroxylamine hydrochloride (88 mg, 0.55 mmol) and 4-dimethylaminopyridine (128 mg, 1.05 mmol) in DCM (8 ml). The mixture was stirred at room temperature for 17 hours. The mixture was washed with 1M HCl and brine, then dried (Na_2SO_4), filtered and concentrated to give the pure product (154 mg, 96%), which appeared as a colourless solid.

^{1}H NMR (400 MHz, $CDCl_3$): δ_H 9.76 (s br, 1H, NH), 7.41–7.32 (m, 5H, ArH), 4.94 (d, *J* = 11.2, 1H, C**H**HPh), 4.86 (d, *J* = 11.2, 1H, CH**H**Ph), 4.17–4.15 (m, 1H, $C^{\alpha}H$), 3.40–3.22 (m, 2H, $C^{\delta}H_2$), 2.40–1.80 (m, 4H, $C^{\beta}H_2$ + $C^{\gamma}H_2$), 1.41 (s, 9H, $C(CH_3)_3$)

^{13}C NMR (101 MHz, $CDCl_3$): δ_C 169.8 (C**C**=O), 156.0 (OC=O), 135.5 (Ar), 129.2 (Ar), 128.6 (Ar), 80.8 (**C**$(CH_3)_3$), 78.2 (**C**H_2Ph), 57.6 ($C^{\alpha}H$), 47.1 ($C^{\delta}H_2$), 28.5 (C(**C**$H_3)_3$), 27.4 (CH_2), 24.8 (CH_2)

Spectroscopic data are consistent with those previously reported.[9]

Boc-L-Pro-NHOH **7**-H

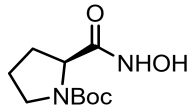

Pd/C (12 mg, 10wt%) was added to a solution of **mw-108** (119 mg, 0.37 mmol) in MeOH (5 mL). The mixture was placed under a hydrogen atmosphere (balloon pressure) and stirred at room temperature for 4.5 hours. The mixture was filtered through celite, then concentrated under reduced pressure. The crude residue was redissolved in ethyl acetate and passed through a silica plug, eluting with EtOAc (R_f = 0.22). Evaporation of the solvent yielded the product (59 mg, 69%) which appeared as a colourless solid. Isolated as a mixture of rotamers.

^{1}H NMR (400 MHz, CD_3OD): δ_H 4.13–4.03 (m, 1H, $C^{\alpha}H$), 3.52–3.37 (m, 2H, $C^{\delta}H_2$), 2.25–1.80 (m, 4H, $C^{\beta}H_2$ + $C^{\gamma}H_2$), 1.46–1.40 (m, 9H ($C(CH_3)_3$).

^{13}C NMR (101 MHz, CD_3OD): (Major rotamer) δ_C 172.5 (C=O), 155.8 (C=O), 81.6 (**C**$(CH_3)_3$), 59. ($C^{\alpha}H$), 47.8 ($C^{\delta}H_2$), 32.4 ($C^{\beta}H_2$), 28.6 (C(**C**$H_3)_3$), 24.7 ($C^{\gamma}H_2$)

(Minor rotamer) δ_C 172.3 (C=O), 156.2 (C=O), 81.3 (**C**$(CH_3)_3$), 59.4 ($C^{\alpha}H$), 47.8 ($C^{\delta}H_2$), 31.4 ($C^{\beta}H_2$), 28.7 (C(**C**$H_3)_3$), 25.3 ($C^{\gamma}H_2$)

HRMS (ESI$^+$, MeOH): Calc. for $C_{10}H_{18}N_2O_4Na$ ($[M+Na]^+$) 253.1159, found 253.1152

Spectroscopic data are consistent with those previously reported.[10]

Bryden's stuff

BL-28a HBr.H-(*R*)-Aib*-OH

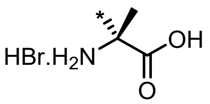

(* = $^{13}CH_3$)

Following an adapted method reported by Clayden and co-workers,[11] potassium hexamethyl disilazide (8.5 M in THF, 8.5 mmol) was cooled to –78 °C. The cold solution was then quickly added (5 × 5.7 mL portions over 3 min) to a stirred solution of (*S*)-2,2-dimethyl-4-methyl-3-(1-naphthoyl)-oxazolidin-5-one[11] (1.15 g, 4.05 mmol) at –78 °C in THF (30 mL). After 4 min, $^{13}CH_3I$ (1.05 mL, 5.74 mmol) was added in one portion. After a further 30 min, HCl (1 M, 15 mL) was added. The reaction mixture was allowed to warm to room temperature, partitioned between water (40 mL) and EtOAc (60 mL), and the organic phase was concentrated. HBr (47% in water, 30 mL) was added, and the mixture was heated to reflux for 18 h then cooled to room temperature, diluted with water (50 mL) and washed with DCM (2 x 20 mL) and Et_2O (20 mL). The aqueous phase was concentrated to dryness, triturated with diethyl ether (10 mL) and dried under reduced pressure to give the crude hydrobromide salt, which was used without further purification.

The optical purity of the salt was determined by first Cbz protection of the amine, then coupling to H-Phe-O^{t}Bu (see below). This gave a mixture of diastereomers, the diastereomeric ratio of which could be quantified by integration of the $^{13}CH_3$ peaks in the ^{13}C NMR spectrum. The d.r. was determined to be 80:20 in this way, therefore HBr.H-(*R*)-Aib*-OH was prepared in an 80:20 (*R*:*S*) ratio.

BL-28 Cbz-Aib*-OH

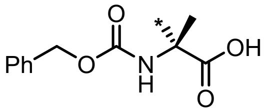

To a solution of HBr.H-(*R*)-Aib*-OH (505 mg, 2.73 mmol) and Na_2CO_3 (868 mg, 8.19 mmol) in water (9.5 mL), a solution of benzyl chloroformate (0.78 mL, 5.46 mmol) in 1,4-dioxane (5.5 mL) was added at 0 °C over 15 min. The mixture was stirred at 0 °C for 1.5 h, before it was allowed to warm to room temperature and stirred for a further 20 h. The reaction mixture was washed with Et_2O (2 x 15 mL), acidified with HCl (conc.) to pH=1, and extracted with EtOAc (2 x 20 mL). The combined organic phases were dried ($MgSO_4$), filtered and concentrated. The crude residue was purified by column chromatography (P.E.:EtOAc, 1:1) to give the title compound (751 mg, 81%) as a colourless solid.

^{1}H NMR (400 MHz, $CDCl_3$): δ_H 7.30–7.40 (m, 5H, ArCH x5), 5.35 (br s, 1H, NH), 5.12 (s, 2H, C**H**$_2$Ph), 1.59 (d, *J* = 4.5, 3H, CH_3), 1.59 (d, *J* = 130.0, 3H, *CH_3)

^{13}C NMR (101 MHz, $CDCl_3$): δ_C 179.5 (C=O), 155.3 (C=O (Cbz)), 136.1 (ArC), 128.5 (ArCH), 128.1 (ArCH), 66.9 (CH_2), 56.3 (d, *J* = 36.5, **C**$^\alpha$-*CH_3), 25.1 (*CH_3)

IR (neat): 3328, 3036, 2989, 2932, 1716, 1683

HRMS (ESI$^-$, DCM): Calc. for $C_{11}{}^{13}CH_{14}NO_4$ ($[M-H]^-$) 237.0961, found 237.0968

Mp 69-71 °C.

BL-T29 (121) Cbz-(*R*)-Aib*-Phe-O^{t}Bu

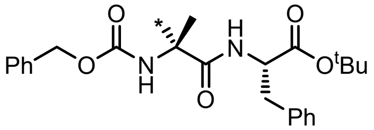

Cbz-Aib*-OH 28 (28 mg, 0.12 mmol) and HOBt hydrate (27 mg, 0.15 mmol) were added to DCM (5 mL) and the solution cooled to 0 °C. EDC (21 µL, 0.12 mmol) was added, and the mixture allowed to warm to room temperature. Once the reaction mixture was homogenous, Phe-OtBu (40 mg, 0.15 mmol) and NEt_3 (41 µL, 0.30 mmol) were added and the solution stirred for 24 h. The mixture was diluted with EtOAc (20 mL), washed with $KHSO_4$ (5%, 2 x 5 mL), $NaHCO_3$ (sat., 2 x 5 mL), brine (5 mL), dried ($MgSO_4$), filtered and concentrated. The crude residue was purified by column chromatography (3:1 P.E.:EtOAc) to give the title compound (39 mg, 75%) as a colourless solid.

^{1}H NMR (500 MHz, $CDCl_3$): δ_H 7.23–7.30 (m, 5H, ArCH x5), 7.07–7.19 (m, 5H, ArCH x5), 6.60 (br s, 1H, NH), 5.31 (br s, 1H, NH), 5.00 (d, *J* = 12.0, 1H, OC**H**HPh), 4.99 (d, *J* = 12.0, 1H, OCH**H**Ph), 4.65 (ddd, *J* = 5.5, 5.5, 5.5, 1H, CH), 3.01 (m, 2H, CC**H**$_2$Ph), 1.41 (d, *J* = 129.0, 3H, *CH_3 major and minor), 1.41 (m, 3H, CH_3 major and minor), 1.33 (s, 9H, $C(CH_3)_3$)

^{13}C NMR (126 MHz, $CDCl_3$): δ_C 173.6 (C=O), 170.4 (C=O), 154.8 (C=O (Cbz)), 136.2 (ArC), 129.5 (ArCH), 128.5 (ArCH), 128.3 (ArCH), 128.1 (ArCH), 128.0 (ArCH), 126.9 (ArC), 82.8 (**C**$(CH_3)_3$), 66.6 (O**C**H_2Ph), 56.8 (d, *J* = 37.0, **C**$^{\alpha}$-*CH_3), 53.6 ($^{\alpha}$CH), 37.9 (C**C**H_2Ph), 27.9 (C(**C**H_3)$_3$), 25.5 (CH_3* major), 25.2 (CH_3* minor)

IR (neat): 3306, 2978, 1724, 1662, 1498

HRMS (ESI$^+$, MeOH): Calc. for $C_{24}{}^{13}CH_{33}N_2O_5$ ($[M+H]^+$) 442.2418, found 442.2410

Mp 117-119 °C

BL-154 HCl.H-(*R*)-Aib*-OEt

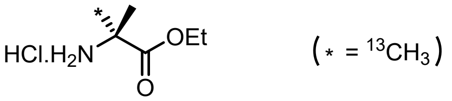

HCl.H-(R)-Aib*OEt was prepared from HBr.H-(*R*)-Aib*-OH (5.29 mmol scale) according to the procedure by Clayden and co-workers[12] to give the pure product (892 mg, >99%) as a pale yellow solid.

^{1}H NMR (500 MHz, CD_3OD): δ_H 4.30 (2H, q, *J* = 7.0, CH_2), 1.57 (3H, d, *J* = 4.5, CH_3), 1.57 (3H, d, J = 130.0, $^{13}CH_3$), 1.33 (3H, t, *J* = 7.5, CH_2C**H**$_3$)

^{13}C NMR (126 MHz, CD_3OD): δ_C 172.7 (C=O), 63.9 (CH_2), 57.7 (d, *J* = 35.5, C$^{\alpha}$), 24.0 (*CH_3 and CH_3), 14.3 (CH_2**C**H_3).

Spectroscopic data are consistent with those previously reported.[12]

BL-155 N_3-Aib$_4$Aib*-OEt

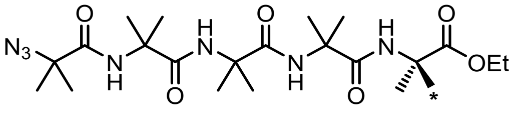

Prepared according to the procedure by Clayden and co-workers[12] (1.47 mmol scale) to give the title compound (377 mg, 51%) as an off-white solid.

^{1}H NMR (400 MHz, $CDCl_3$): δ 7.28 (br s, 1H, NH), 7.11 (br s, 1H, NH), 6.86 (br s, 1H, NH), 6.12 (br s, 1H, NH), 4.14 (q, *J* = 7.0, 2H, CH_2), 1.55 (s, 6H, CH_3 x2), 1.51 (m, 3H, C**H**$_3$-C-*CH_3), 1.50 (d, *J* = 129.0, 3H, *CH_3), 1.50 (s, 6H, CH_3 x2), 1.49 (s, 6H, CH_3 x2), 1.43 (s, 6H, CH_3 x2), 1.23 (t, *J* = 7.0, 3H, C**H**$_3$$CH_2$).

^{13}C NMR (101 MHz, $CDCl_3$): δ 174.8 (C=O), 174.0 (C=O), 172.82 (C=O), 172.81 (C=O), 172.3 (C=O), 64.0 (C^α), 60.6 (CH_2), 56.9 (C^α), 56.81 (C^α), 56.78 (C^α), 55.7 (d, *J* = 37.0, C^α-*CH_3), 25.4 (CH_3), 25.3 (CH_3), 24.9 (CH_3), 24.8 (*CH_3), 24.3 (CH_3), 14.1 (**C**H_3CH_2)

Spectroscopic data are consistent with those previously reported.[12]

BL-231 H_2N-Aib_4Aib*-OEt

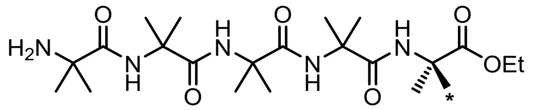

Pd/C (35 mg, 10wt%) was added to a solution of N_3-Aib_4Aib*-OEt (357 mg, 0.72 mmol) in MeOH (10 mL). The mixture was placed under a hydrogen atmosphere (balloon pressure) and stirred at room temperature for 3 d. The mixture was filtered through a celite pad with charcoal on top, eluting with MeOH, and the solution was concentrated under reduced pressure to give the title compound (339 mg, >99%) as a colourless solid which was used without further purification.

^{1}H-NMR (400 MHz, CD_3OD): δ_H 4.11 (q, *J* = 7.0, 2H, CH_2), 1.48 (d, *J* = 4.5, 3H, CH_3), 1.48 (d, *J* = 128.5, 3H, *CH_3), 1.47 (s, 6H, CH_3 x2), 1.44 (s, 6H, CH_3 x2), 1.38 (s, 6H, CH_3 x2), 1.33 (s, 6H, CH_3 x2), 1.23 (t, *J* = 7.0, 3H, C**H**$_3$$CH_2$)

^{13}C-NMR (101 MHz, CD_3OD): δ_C 179.8 (C=O), 176.8 (C=O), 176.6 (C=O), 176.4 (C=O), 176.3 (C=O), 62.0 (CH_2), 57.8 (C^α), 57.7 (C^α), 57.4 (C^α), 57.0 (d, *J* = 37.0, C^α-*CH_3), 55.7 (C^α), 28.4 (CH_3), 25.7 (CH_3), 25.4 (CH_3), 25.2 (*CH_3), 24.9 (CH_3), 14.4 (**C**H_3CH_2)

BL-215 6-(aminomethyl)nicotinic acid

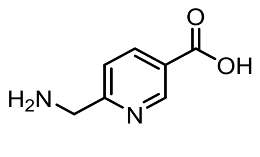

Prepared according to the procedure reported by Zhang et al.[13] (1 mmol scale) to give the title compound (151 mg, >99%) as a colourless solid.

^{1}H NMR (400 MHz, D_2O): δ_H 8.91 (m, 1H, ArH), 8.21 (dd, *J* = 8.0, 2.0, 1H, ArH), 7.49 (d, *J* = 8.0, 1H, ArH), 4.29 (s, 2H, CH_2)

^{13}C NMR (101 MHz, D_2O): δ_C 173.5 (C=O), 155.3 (Ar), 150.2 (Ar), 139.0 (Ar), 132.5 (Ar), 123.0 (Ar), 44.0 (CH_2)

Spectroscopic data are consistent with those previously reported.[13]

BL-121 methyl 6-(aminomethyl)nicotinate hydrochloride

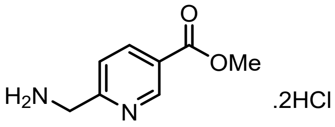

Prepared according to a modified version of the procedure reported by Zhang et al.[13] **BL-215** (152 mg, 1 mmol) was dissolved in MeOH (10 mL) and thionyl chloride (0.3 mL) was added dropwise. The resulting solution was heated under reflux for 16 h. The mixture was concentrated and Et_2O was added. The precipitate formed was filtered to give the title compound (163 mg, 98%) as a colourless solid.

^{1}H NMR (400 MHz, CD_3OD): δ_H 9.19 (d, *J* = 2.0, 1H, ArH), 8.43 (dd, *J* = 8.0, 2.0, 1H, ArH), 7.62 (d, *J* = 8.0, 1H, ArH), 4.40 (s, 2H, CH_2), 3.96 (s, 3H, OCH_3)

^{13}C NMR (101 MHz, CD_3OD): δ_C 166.6 (C=O), 157.9 (Ar), 151.2 (Ar), 139.8 (Ar), 127.5 (Ar), 123.6 (Ar), 53.2 (OCH_3), 44.0 (CH_2)

IR (neat): 2915, 2441, 1741, 1644

HRMS (ESI$^+$, MeOH): Calc. for $C_8H_{11}N_2O_3$ $[M+H]^+$ 167.0816, found 167.0818.

Mp >222 °C (decomp.).

BL-122 Methyl 6-((bis(quinolin-2-ylmethyl)amino)methyl)nicotinate

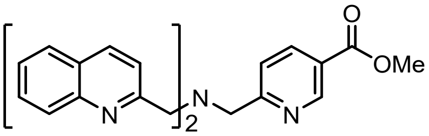

2-(Bromomethyl)-quinoline (888 mg, 4 mmol) and *N*,*N*-diisopropylethylamine (1.74 mL, 10 mmol) were dissolved in THF (15 mL). **BL-121** (405 mg, 2 mmol) was added and the resulting mixture stirred for 3 d. The reaction mixture was filtered and concentrated. The crude reaction product was purified by alumina column chromatography (0.4% NEt_3 in EtOAc) to give the title compound (511 mg, 57%) as a yellow solid.

^{1}H NMR (400 MHz, $CDCl_3$): δ_H 9.13 (dd, *J* = 2.5, 1.0, 1H, PyCH) 8.26 (dd, *J* = 8.0, 2.0, 1H, PyCH), 8.15 (d, *J* = 8.5, 2H, QuinCH x2), 8.06 (d, *J* = 8.5, 2H, QuinCH x2), 7.80 (d, *J* = 8.5, 2H, QuinCH x2), 7.73 (d, *J* = 8.5, 2H, QuinCH x2), 7.72 (dd, *J* = 8.0, 0.5, 1H, PyCH), 7.72 (m, 2H, QuinCH x2), 7.52 (ddd, *J* = 8.5, 7.0, 1.5, 2H, QuinCH x2), 4.10 (s, 4H, CH_2-Quin x2), 4.04 (s, 2H, CH_2-Py), 3.94 (s, 3H, OCH_3)

^{13}C NMR (101 MHz, $CDCl_3$): δ_C 165.8 (C=O), 164.1 (PyC), 159.7 (QuinC), 150.3 (PyCH), 147.6 (QuinC), 137.4 (PyCH), 136.5 (QuinCH), 129.5 (QuinCH), 129.1 (QuinCH), 127.5 (QuinCH), 127.3 (QuinC), 126.3 (QuinCH), 124.4 (PyC), 122.7 (PyCH), 121.0 (QuinCH), 61.1 (CH_2-Quin), 60.2 (CH_2-Py), 52.3 (OCH_3)

IR (neat) = 3058, 2947, 2814, 1717, 1616, 1595

HRMS (ESI$^+$, DCM): Calc. for $C_{28}H_{25}N_4O_2$ ($[M+H]^+$) 449.1973, found 449.1964

Mp 133-135 °C

BL-156 6-((bis(quinolin-2-ylmethyl)amino)methyl)nicotinic acid

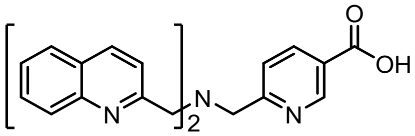

6-((Bis(quinolin-2-ylmethyl)amino)methyl)nicotinic acid **BL-122** (468 mg, 1.04 mmol) was dissolved in MeOH (15 mL), then a solution of KOH (1 M, 5 mL) was added and the resulting solution heated under reflux for 4 h. After being allowed to cool to room temperature, the MeOH was removed in vacuo and HCl (1 M, aq.) was added until the pH=5-6. Brine (2 mL) was added, and the solution was extracted with DCM (2 x 30 mL). The combined organic phases were concentrated in vacuo to give the title compound (449 mg, 99%) as a yellow solid.

^{1}H NMR (300 MHz, $CDCl_3$): δ_H 9.21 (d, *J* = 1.5, 1H, PyCH), 8.25 (m, 1H, PyCH), 8.21 (m, 4H, QuinCH x4), 7.81 (m, 4H, QuinCH x4), 7.72 (m, 2H, QuinCH x2), 7.61 (d, *J* = 8.0, 1H, PyCH), 7.53 (m, 2H, QuinCH x2), 4.27 (s, 4H, CH_2-Quin x2), 4.10 (s, 2H, CH_2-Py)

^{13}C NMR (75 MHz, $CDCl_3$): δ_C 167.4 (C=O), 162.0 (QuinC), 159.5 (PyC), 150.6 (PyCH), 146.9 (QuinC), 137.9 (PyCH), 137.2 (QuinCH), 129.9 (QuinCH), 128.3 (QuinCH), 127.52 (QuinCH), 127.49 (QuinC), 126.6 (QuinCH), 126.8 (PyC), 123.2 (PyCH), 121.6 (QuinCH), 59.8 (CH_2-Quin), 59.3 (CH_2-Py)

IR (neat): 2825, 2451, 1923, 1704, 1596, 1504

HRMS (ESI$^+$, DCM): Calc. for $C_{27}H_{21}N_4O_2$ ($[M+H]^+$) 433.1670, found 433.1669

Mp 96-98 °C

BL-157 BQPA-3-(CO-Aib$_4$Aib*-OEt)

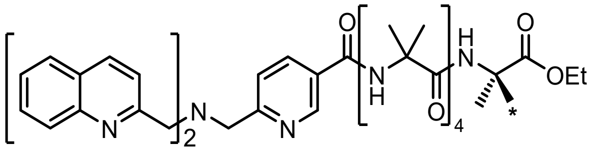

BL-156 (352 mg, 0.81 mmol), HOBt hydrate (148 mg, 0.84 mmol), triethylamine (0.26 mL, 1.52 mmol) and **BL-231** (319 mg, 0.67 mmol) were dissolved in DCM (8 mL). EDC hydrochloride (161 mg, 0.84 mmol) was added, and the reaction mixture was stirred for 48 h. DCM (50 mL) was added and the organic phase washed with H_2O (2 x 10 mL), dried (Na_2SO_4), filtered and concentrated. The crude residue was purified by column chromatography on alumina (1-5% EtOH in DCM) to give the title compound (290 mg, 49%) as a pale-yellow solid.

^{1}H-NMR (400 MHz, $CDCl_3$): δ_H 9.00 (s, 1H, PyCH), 8.16 (d, *J* = 8.5, 2H, QuinCH x2), 8.16 (m, 1H, PyCH), 8.06 (d, *J* = 8.5, 2H, QuinCH x2), 7.74 (m, 7H, QuinCH x6 and PyCH), 7.60 (br s, 1H, NH), 7.52 (dd, *J* = 7.5, 7.5, 2H, QuinCH x2), 7.45 (br s, 1H, NH), 7.35 (br s, 1H, NH), 7.20 (br s, 1H, NH), 6.55 (br s, 1H, NH), 4.09 (s, 4H, CH_2-Quin x2), 4.05 (m, 2H, CH_2CH_3, 4.05 (s, 2H, CH_2-Py), 1.61 (s, 6H, CH_3 x2), 1.50 (d, *J* = 129.0, 3H, *CH_3), 1.50 (m, 15H, CH_3 x5), 1.44 (s, 6H, CH_3 x2), 1.18 (t, *J* = 7.0, 3H, $CH_2\mathbf{CH_3}$)

^{13}C-NMR (101 MHz, $CDCl_3$): δ_C 175.0 (C=O), 174.6 (C=O), 174.1 (C=O), 173.8 (C=O), 173.3 (C=O), 166.2 (C=O-Py), 159.5 (QuinC), 147.8 (PyCH), 147.6 (QuinC), 136.6 (QuinCH), 135.7 (PyCH), 129.6 (QuinCH), 129.0 (QuinCH), 127.5 (QuinCH), 127.4 (QuinC), 127.2 (PyC), 126.4 (QuinCH), 123.1 (PyCH), 121.0

(QuinCH), 61.0 (CH_2-Quin), 60.6 (CH_2-Py), 59.9 (**C**H_2CH_3), 57.6 (C^α), 57.0 (C^α), 56.79 (d, *J* = 40.5, **C**$^\alpha$-*CH_3), 56.77 (C^α), 56.6 (C^α), 25.4 (CH_3), 25.2 (CH_3), 25.1 (CH_3), 24.9 (*CH_3), 24.8 (CH_3), 14.1 (CH_2CH_3) One carbon (PyC) could not be located

IR (neat): 3329, 3044, 2983, 2936, 1722, 1663, 1642, 1597, 1528, 1506, 1467, 1454, 1428

HRMS (ESI$^+$, MeOH): Calc. for C48 13CH61N9O7Na ([M+Na]$^+$) 911.4625, found 911.4620.

Mp 177-180 °C.

BL-232 BQPA-3-(CO-Aib$_4$Aib*-OH)

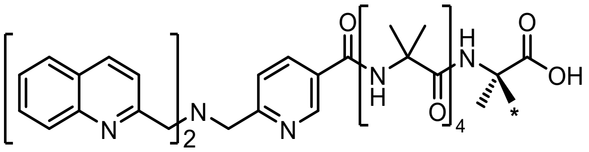

157 (149 mg, 0.17 mmol) was dissolved in MeOH (5 mL) and KOH (1 M, 1 mL) and the resulting solution stirred at 60 °C for 16 h. After being allowed to cool to room temperature, the MeOH was removed in vacuo and HCl (1 M) was added until the pH = 5-6. Water (3 mL) was added, and the solution was extracted with $CHCl_3$ (2 x 15 mL). The combined organic phases were concentrated in vacuo to give the title compound (134 mg, 93%) as a light yellow solid.

^{1}H-NMR (400 MHz, $CDCl_3$): δ_H 9.22 (s, 1H, PyCH), 8.50 (br s, 1H, NH), 8.36 (d, *J* = 7.5, 1H, PyCH), 8.17 (d, *J* = 8.5, 2H, QuinCH x2), 8.10 (br s, 1H, NH), 8.09 (d, *J* = 8.5, 2H, QuinCH x2), 7.86 (br s, 1H, NH), 7.80 (d, *J* = 8.0, 2H, QuinCH x2), 7.73 (d, *J* = 7.5, 2H, QuinCH x2), 7.72 (br s, 1H, NH), 7.69 (d, *J* = 8.5, 2H, QuinCH x2), 7.69 (m, 1H, PyCH), 7.60 (br s, 1H, NH), 7.53 (dd, *J* = 7.5, 7.5, 2H, QuinCH x2), 4.27 (s, 4H, CH_2-Quin x2), 4.18 (s, 2H, CH_2-Py), 1.61 (s, 6H, CH_3 x2), 1.50 (s, 12H, CH_3 x4), 1.42 (d, *J* = 130.5, 3H, *CH_3), 1.42 (m, 3H, C**H**$_3$-C-*CH_3), 1.41 (s, 6H, CH_3 x2)

^{13}C-NMR (101 MHz, $CDCl_3$): δ_C 176.8 (C=O), 176.6 (C=O), 175.5 (C=O), 175.4 (C=O), 175.0 (C=O), 166.5 (C=O-Py), 148.9 (PyCH), 146.8 (PyC), 137.4 (QuinCH), 136.5 (PyCH), 130.1 (QuinCH), 128.5 (QuinCH), 128.4 (PyC), 127.6 (QuinC), 127.4 (QuinCH), 126.8 (QuinCH), 122.9 (PyCH), 120.9 (QuinCH), 60.0 (CH_2-Quin), 59.6 (CH_2-Py), 57.6 (C^α), 56.9 (d, *J* = 37.0, **C**$^\alpha$-*CH_3), 56.8 (C^α), 56.72 (C^α), 56.67 (C^α), 24.9 (*CH_3 and all CH_3). Two carbons (QuinC x2) could not be located

IR (neat): 3305, 3050, 2985, 2938, 1649, 1599, 1532, 1507, 1466, 1455

HRMS (ESI$^+$, MeOH): Calc. for $C_{46}{}^{13}CH_{58}N_9O_7$ ([M+H]$^+$) 861.4493, found 861.4457

Mp 224-226 °C.

BL-T158 (450) BQPA-3-(CO-Aib4Aib*-NHMe) **8**

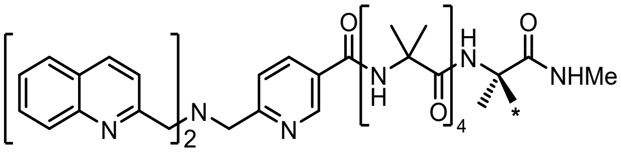

BL-232 (119 mg, 0.14 mmol), HOBt hydrate (32 mg, 0.18 mmol), triethylamine (87 μL, 0.62 mmol) and methylamine hydrochloride (23 mg, 0.35 mmol) were dissolved in DCM (5 mL) and then EDC hydrochloride (29 mg, 0.15 mmol) was added. The resulting solution was stirred for 16 h, then the solvent removed in vacuo and the residue re-dissolved in $CHCl_3$/IPA (3:1, 30 mL). The organic phase was washed with H_2O (5 mL), dried (Na_2SO_4), filtered and concentrated. The crude residue was purified by column chromatography on alumina (0.5-5% MeOH in DCM) to give the title compound (91 mg, 75%) as yellow solid.

^{1}H-NMR (500 MHz, CDCl$_3$) δ_H 8.99 (br s, 1H, PyCH), 8.16 (m, 1H, PyCH), 8.16 (d, *J* = 8.5, 2H, QuinCH x2), 8.06 (d, *J* = 8.5, 2H, QuinCH x2), 7.80 (d, *J* = 8.5, 2H, QuinCH x2), 7.77 (d, *J* = 6.5, 1H, PyCH), 7.74 (d, *J* = 8.5, 2H, QuinCH x2), 7.71 (ddd, *J* = 8.5, 7.0, 1.5, 2H, QuinCH x2), 7.67 (br s, 1H, NH), 7.67 (br s, 1H, NH), 7.53 (ddd, *J* = 8.0, 7.0, 1.0, 2H, QuinCH x2), 7.39 (q, *J* = 4.5, 1H, N**H**CH$_3$), 7.31 (br s, 1H, NH), 7.24 (br s, 1H, NH), 6.57 (br s, 1H, NH), 4.10 (s, 4H, CH$_2$-Quin x2), 4.06 (s, 2H, CH$_2$-Py), 2.75 (d, *J* = 4.5, 3H, NHC**H**$_3$), 1.61 (s, 6H, CH$_3$ x2), 1.53 (s, 6H, CH$_3$ x2), 1.51 (d, *J* = 4.5, 3H, C**H**$_3$-C$^\alpha$-*CH$_3$), 1.51 (d, *J* = 129.0, 3H, *CH$_3$), 1.48 (s, 6H, CH$_3$ x2), 1.43 (s, 6H, CH$_3$ x2)

^{13}C-NMR (126 MHz, CDCl$_3$) δ_C 176.1 (C=O), 175.6 (C=O), 174.4 (C=O), 174.0 (C=O), 173.5 (C=O), 166.3 (C=O-Py), 163.8 (PyC), 159.6 (QuinC), 147.7 (PyCH), 147.6 (QuinC), 136.5 (QuinCH), 135.5 (PyCH), 129.6 (QuinCH), 129.1 (QuinCH), 127.5 (QuinCH), 127.4 (QuinC), 127.0 (PyC), 126.4 (QuinCH), 123.2 (PyCH), 121.0 (QuinCH), 61.0 (CH$_2$-Quin), 59.9 (CH$_2$-Py), 57.6 (C$^\alpha$), 57.2 (d, *J* = 37.5, **C**$^\alpha$-*CH$_3$), 57.0 (C$^\alpha$), 56.9 (C$^\alpha$), 56.8 (C$^\alpha$), 26.5 (NH**C**H$_3$), 25.9 (*CH$_3$ and CH$_3$), 25.2 (CH$_3$), 25.1 (CH$_3$), 24.9 (CH$_3$)

IR (neat): 3303, 2983, 2933, 1643, 1597, 1533, 1505, 1468, 1427

HRMS (ESI$^+$, MeOH): Calc. for $C_{47}{}^{13}CH_{60}N_{10}O_6Na$ ([M+Na]$^+$) 896.4623, found 896.4627

Mp 154-156 °C.

<u>**BL-T159 (463)** [Zn(BQPA-3-(CO-Aib$_4$Aib*-NHMe))(ClO$_4$)$_2$] Zn(**8**).2ClO$_4$</u>

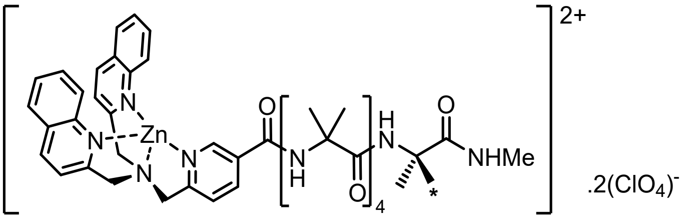

A solution of zinc perchlorate hexahydrate (82 mg, 0.22 mmol) in MeOH (2 mL) was added dropwise to a suspension of **BL-T158** (187 mg, 0.22 mmol) in MeOH (3 mL). The mixture was stirred at room temperature for 10 min, then Et_2O (18 mL) was added. After stirring for a further hour, the mixture was filtered and the precipitate washed with Et_2O to give the title compound (190 mg, 74%) as an off-white solid.

^{1}H-NMR (400 MHz, CD$_3$OD) δH 9.27 (br s, 1H, PyCH), 8.56 (dd, *J* = 8.0, 2.0, 1H, PyCH), 8.55 (m, 2H, QuinCH x2), 8.53 (d, *J* = 8.5, 2H, QuinCH x2), 8.02 (br s, 1H, NH), 8.02 (d, *J* = 7.5, 2H, QuinCH x2), 7.94 (dd, *J* = 7.5, 7.5, 1H, QuinCH x2), 7.76 (br s, 1H, NH), 7.74 (d, *J* = 8.5, 1H, PyCH), 7.71 (dd, *J* = 7.5, 7.5, 2H, QuinCH x2), 7.62 (br s, 1H, NH), 7.60 (d, *J* = 8.5, 2H, QuinCH x2), 4.80 (d, *J* = 17.5, 2H, C**H**H-Quin), 4.75 (d, *J* = 17.5, 2H, CH**H**-Quin), 4.70 (s, 2H, CH$_2$-Py), 2.75 (s, 3H, NHC**H**$_3$), 1.53 (s, 6H, CH$_3$ x2), 1.50 (d, *J* = 4.5, 3H, C**H**$_3$-C$^\alpha$-*CH$_3$), 1.50 (d, *J* = 129.0, 3H, *CH$_3$), 1.48 (s, 6H, CH$_3$ x2), 1.41 (s, 6H, CH$_3$ x2), 1.34 (s, 6H, CH$_3$ x2).

^{13}C-NMR (101 MHz, CD_3OD) δC 178.3 (C=O), 178.1 (C=O), 177.0 (C=O), 176.2 (C=O), 166.1 (C=O-Py), 159.0 (QuinC), 158.6 (PyC), 149.1 (PyCH), 146.2 (QuinC), 142.7 (QuinCH), 140.7 (PyCH), 133.2 (QuinCH), 132.6 (PyC), 130.23 (QuinC), 130.15 (QuinCH), 129.2 (QuinCH), 126.8 (QuinCH), 125.6 (PyCH), 122.6 (QuinCH), 62.5 (CH_2-Quin), 61.2 (CH_2-Py), 58.5 (C^{α}), 58.4 (d, *J* = 37.5, **C**$^{\alpha}$-*CH_3), 58.1 (C^{α}), 57.9 (C^{α}), 57.8 (C^{α}), 26.6 (NH**C**H_3), 26.0 (*CH_3 and CH_3), 25.5 (CH_3), 25.4 (CH_3), 25.1 (CH_3). One CO could not be located.

IR (neat): 3301, 2971, 2934, 1649, 1604, 1533, 1517, 1467, 1436, 1098.

LRMS (ESI$^+$, MeOH): 469 (100%, [^{64}Zn(BQPA-3-(CO-Aib$_4$Aib*-NHMe))]$^{2+}$), 470 (45%, [^{66}Zn(BQPA-3-(CO-Aib$_4$Aib*-NHMe))]$^{2+}$), 471 (35%, [^{68}Zn(BQPA-3-(CO-Aib$_4$Aib*-NHMe))]$^{2+}$).

Mp >219 °C (decomp.).

S3. CD spectroscopy methodology

S3.1 Preparation of stock solutions

Stock solutions of non-hygroscopic solid compounds were prepared as 2.50 mM solutions by weighing out the appropriate mass of compound in a volumetric flask (dried by heat gun under vacuum), then dissolving in anhydrous[i] MeCN. For liquid compounds (NEt_3, TfOH, $^tBuP_1(dma)_3$) or hygroscopic solids (Boc-D-Pro.TBA), a small, arbitrary amount of compound was weighed out in a pre-weighed, stoppered volumetric flask (to minimise compound evaporation or water absorption) and dissolved in MeCN to give a solution of arbitrary, but known, concentration. An aliquot of this solution was then diluted to 5.00, 2.50 or 1.25 mM by the addition of the appropriate volume of MeCN, as required.

S3.2 Base titrations and formula for [θ]

An initial sample (c_o = 0.25 mM) was prepared in a 1 mm path length quartz cuvette from Zn(BQPA).2ClO_4 (20 µL, 2.50 mM, 50 nmol) and ligand(s) (20[ii] µL each, 2.50 mM, 50 nmol), made up to 200 µL with MeCN, and its CD spectrum (200–270 nm) was recorded. Then, NEt_3 (2.50 mM) was added in 10 µL (25 nmol, 0.5 eq.) increments and the sample gently shaken; the CD spectrum was recorded after each addition.

During such titration experiments, the sample was diluted after each addition of titrant. It was therefore necessary to convert the raw ellipticity output, θ, into molar values to account for this concentration change. The *molar ellipticity*, $[\theta]_\lambda$, was calculated using the following formula:

$$[\theta]_\lambda = \frac{\theta_\lambda}{cl}$$

[i] Anhydrous MeCN was used for all stock solutions and titration experiments.
[ii] Except (+)-TADDOL-PONHTf, see section S3.3

Where θ_λ is the ellipticity measured at wavelength λ, c is the concentration of Zn(BQPA) in the sample and l is the path length of the cuvette. In practice, it is more convenient to rewrite c in terms of Zn(BQPA) stock solution concentration:

$$[\theta]_\lambda = \frac{V\theta_\lambda}{vc'l}$$

Where c' is the concentration of the Zn(BQPA) stock solution, V is the total volume of the sample and v is the volume of Zn(BQPA) stock solution used in the sample.

S3.3 Base titration to estimate concentration of (+)-TADDOL-PONHTf stock solution

A base titration was performed using a freshly prepared[iii] stock solution of (+)-TADDOL-PONHTf (**5**-H) with a nominal concentration of 2.50 mM. An initial sample (c_o = 0.25 mM) was prepared from Zn(BQPA).2ClO_4 (20 µL, 2.50 mM, 50 nmol) and **5**-H (20 µL, "2.50" mM) in MeCN (160 µL, 200 µL total), and its CD spectrum was recorded. A solution of NEt_3 (1.25 mM) was added in 10 µL (12.5 nmol, 0.25 eq.) increments and the CD spectrum recorded after each addition. Maximum signal magnitude was expected at 1 equivalent of base as **5**-H should be completely deprotonated at this point, while excess base would displace **5**$^-$ from the metal centre, reducing signal magnitude. However, the signal maximum was observed at 0.5 equivalents of base, suggesting that the true concentration of **5**-H was less than 0.25 mM (Fig. S1). This was most likely due to the presence of metal salts $\mathbf{5}_xM_y$ in the sample of **5**-H (see main article).

Due to the extremely large pK_a difference between **5**-H and $HNEt_3^+$ (8.5 vs. 18.83, main article Table 1), the titration curve from 0 to 1 equivalent of base should be approximately linear, assuming that the binding constant of **5**$^-$ is not so small that the binding curve deviates from the typical tight-binding curve. Similarly, the signal should decay approximately linearly immediately after the signal maximum, due to displacement of **5**$^-$ by excess NEt_3. It should therefore be possible to estimate the position of this maximum by extrapolating the linear segments of the titration curve and finding the intersection.

Linear trendlines were extrapolated from the first two groups of three data points (0, 0.25, 0.5 and 0.75, 1, 1.25) either side of the hypothetical signal maximum. Solving the two simultaneous equations gave the coordinates of the intersection as (0.61, -13,394). Thus, the true concentration of **5**-H in this sample was estimated to be 0.25*0.61 = 0.15 mM. All subsequent titration experiments involving **5**-H were performed using freshly prepared **5**-H stock solution titrated in this manner prior to use. Where an experiment called for 20 µL of ligand to be used in a sample, 20/x µL was used instead, where x was the x-coordinate of the hypothetical signal maximum.

[iii] A precipitate formed within a couple of days in stock solutions of **5**-H under ambient conditions. Storage in the freezer slowed, but did not halt, this process.

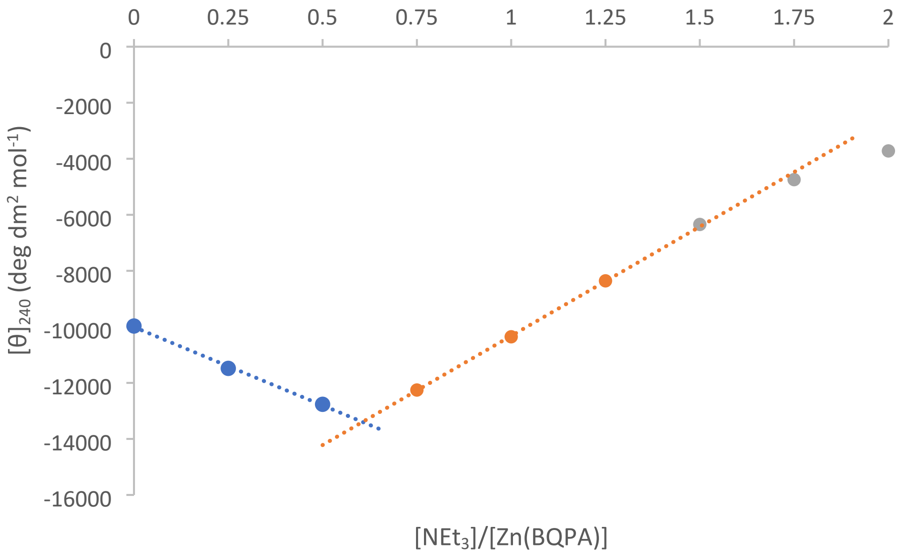

Figure S1: Molar ellipticities at 240 nm of Zn(**1**) (c_0 = 0.25 mM) with **5**-H ("0.25 mM" nominal concentration) and NEt_3 (0.0 → 2.0 eq.)

S3.4 Base/acid cycling experiments

An initial sample (c_o = 0.25 mM) was prepared in a 1 mm path length quartz cuvette from Zn(BQPA).2ClO_4 (20 µL, 2.50 mM, 50 nmol) and *n* ligands (20[iv] µL each, 2.50 mM, 50 nmol), made up to 200 µL with MeCN, and its CD spectrum (200–270 nm) was recorded. Base (NEt_3 or ^{t}BuP_1(dma)$_3$, 5.0 mM) was added in 10 µL (50 nmol, 1 eq.) increments up to *n* equivalents of base. Then, for *n* = 1 or 2, TfOH (10 µL, 5.0 mM, 50 nmol) was added followed by base (10 µL, 5.0 mM, 50 nmol). For *n* = 3, TfOH (5.0 mM) was added in 10 µL (50 nmol, 1 eq.) increments up to 2 equivalents of acid, then base (5.0 mM) was added in 10 µL (50 nmol, 1 eq.) increments up to 2 equivalents of base. This process was repeated as desired, with the CD spectrum recorded after each addition of acid or base.

S3.5 Binding constant measurement titration

An initial sample (c_o = 0.25 mM) was prepared in a 1 mm path length quartz cuvette from Zn(BQPA).2ClO_4 (20 µL, 2.50 mM, 50 nmol) and MeCN (180 µL, 200 µL total), and its CD spectrum (200-270 nm) was recorded. Tetrabutylammonium salt **2**.TBA or **4**.TBA (1.25 mM) was added in 10 µL (12.5 nmol, 0.25 eq.) increments up to 2.25 eq., with the CD spectrum recorded after each addition.

[iv] Except (+)-TADDOL-PONHTf, see section S3.3

S4. Miscellaneous CD spectra

S4.1 Ligand spectra in the absence of Zn(BQPA)

CD spectra of 0.25 mM solutions of **2**-H, **4**-H, **5**-H and their tetrabutylammonium (TBA) salts (or triethylammonium salt in the case of **5**-H) in MeCN were recorded (Fig. S2), to prove that any pH-induced changes in the observed CD spectrum were solely due to ligand binding or exchange at the Zn(**1**) receptor complex. All of these ligand species had molar ellipticities of around 0 at 240 nm, the characteristic output wavelength of Zn(**1**), so any structural changes in the ligands had negligible contribution to the overall CD spectrum of the reported CD switching systems.

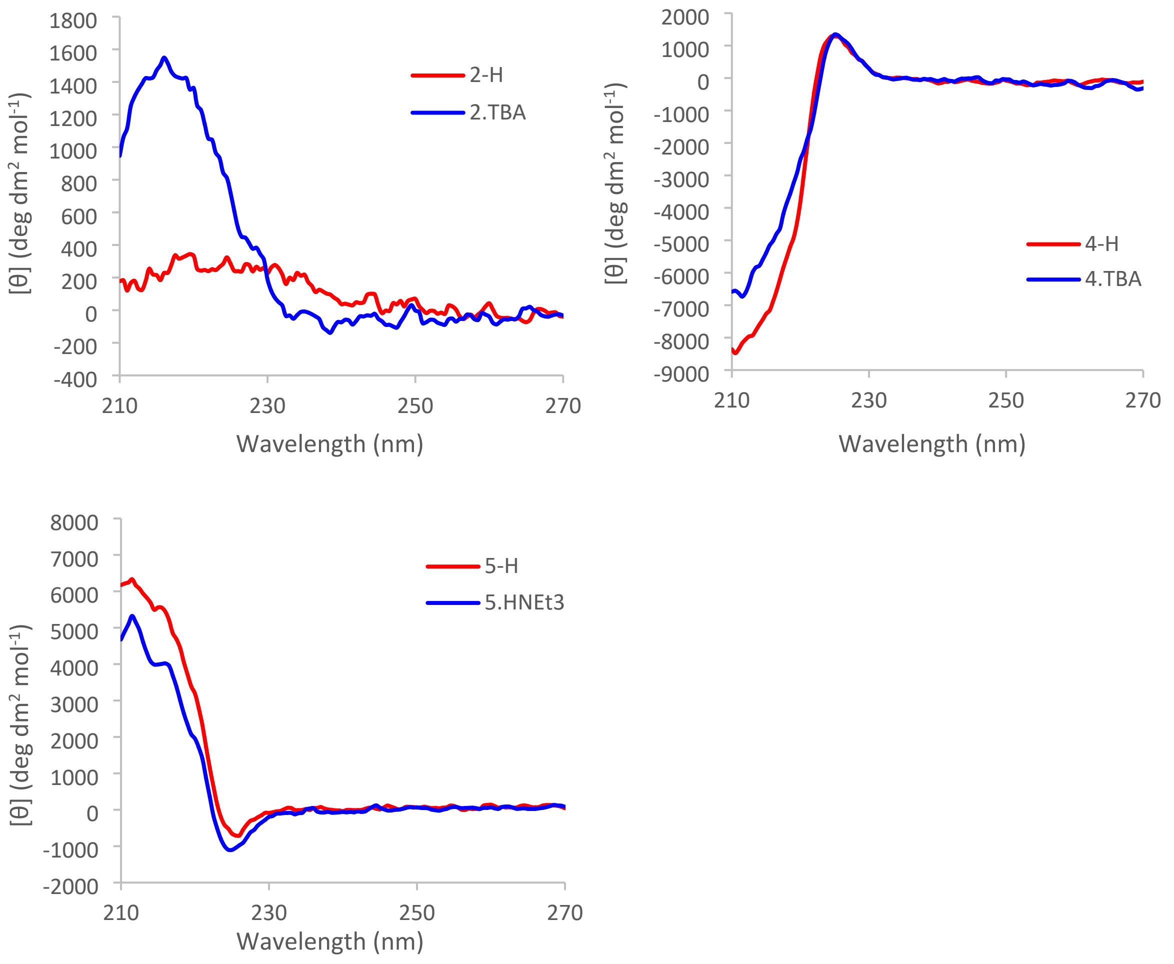

Figure S2: CD spectra of **(a)** **2**-H and **2**.TBA; **(b)** **4**-H and **4**.TBA; **(c)** **5**-H and **5**.$HNEt_3$ (all c = 0.25 mM) in MeCN

S4.2 Unsuccessful lower acidity ligands

Titration of an equimolar 0.25 mM solution of hydroxamic acid **7**-H and Zn(BQPA).$2ClO_4$ with NEt_3 revealed an almost complete absence of a positive signal at 240 nm expected for the Boc-L-prolyl backbone (Fig. S3a). This was not due to a lack of binding since the negative lobe was still present at 232 nm. Hydroxamic acids function as bidentate metal chelators in nature,[14] so bidentate binding with Zn(BQPA) could have had a significant effect on the optical properties of the complex. The zinc centre

must either become six-coordinate by accommodating the extra lone pair or remain five-coordinate by having one of the BQPA groups displaced by **7**⁻. Both possibilities would affect the orientation of the quinoline chromophores around the zinc centre.

The base titration was also performed with thioamide **6**-H, but using triazabicyclodecene (TBD) as a more powerful base (pK_a(MeCN) = 26.02, cf. 18.83 for NEt_3).[15] **6**-H gave a very weak positive signal at 240 nm upon addition of TBD (Fig. S3b). The signal magnitude reached a maximum of only 1000 deg dm^2 mol^{-1} at 1 eq. of base, only 13% of the maximum signal magnitude achieved with 1 eq. Boc-D-Pro-OH, which had the same chiral backbone. Further addition of TBD caused a reduction in signal magnitude, indicating that deprotonation of the ligand was likely complete around 1 eq. of base.

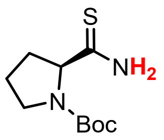

6-H

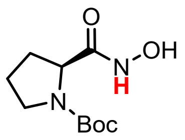

7-H

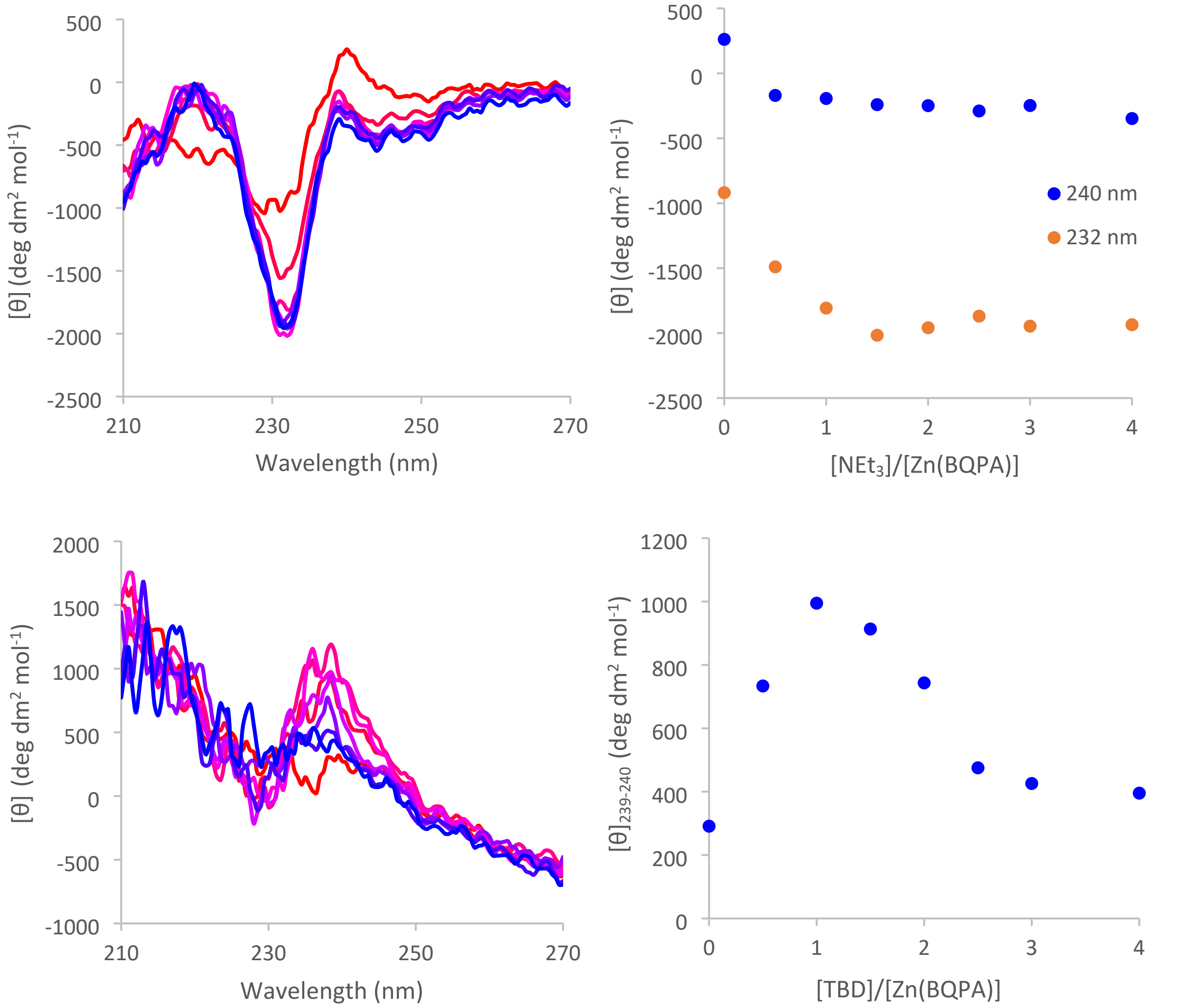

Figure S3: CD spectra of Zn(**1**) (c_0 = 0.25 mM) with **(a) 7**-H (1 eq.) or **(b) 6**-H (1 eq.) in MeCN with addition of NEt_3 (**0.0** → **4.0** eq.) in 0.5 eq. increments. For **7**-H, the $[\theta]_{232}$ and $[\theta]_{240}$ data points were plotted against [NEt_3]/[Zn(BQPA)], while for **6**-H, the average of the $[\theta]_{239}$, $[\theta]_{239.5}$ and $[\theta]_{240}$ nm data points were plotted against [TBD]/[Zn(BQPA)].

S5. NMR titration methodology

Monitoring the binary pH switch by ^{13}C-NMR

Stock solutions of Zn(**8**).2ClO_4 (0.015 M), Boc-L-Pro-OH **9**-H (0.075 M), NEt_3 (0.45 M) and TFA (0.45 M) were made up in MeCN-d_3. The metal complex (500 µL, 7.5 µmol) and **9**-H (120 µL, 9.0 µmol) stock solutions were added to an NMR tube and the ^{1}H and ^{13}C-NMR spectra acquired. The spectra were then reacquired after addition of NEt_3 (21 µL, 9.5 µmol), then TFA (22 µL, 9.9 µmol), then NEt_3 (23 µL, 10.4 µmol), then TFA (26.4 µL, 11.9 µmol), then NEt_3 (26.2 µL, 11.8 µmol).

Monitoring the ternary pH switch by ^{13}C- and ^{31}P-NMR

Stock solutions of the metal complex (0.015 M), **9**-H (0.075 M), NEt_3 (0.825 M) and $HClO_4$ (0.825 M) were made up in MeCN-d_3. The metal complex (500 µL, 7.5 µmol) and Boc-Pro-OH (110 µL, 8.25 µmol) stock solutions, along with (*S*)-TPSP **10**-H (7.14 mg, 8.25 µmol) were added to an NMR tube and the ^{1}H, ^{31}P and ^{13}C-NMR spectra acquired. The spectra were then reacquired following each addition of two additions of triethylamine (10 µL, 8.25 µmol), then two additions of $HClO_4$ (10 µL, 8.25 µmol). For subsequent additions, 10 µL of triethylamine or $HClO_4$ were first added and the spectra reacquired. If the system had not switched fully over to the desired state, further base/acid was added in 2.0 µL (1.7 µmol) increments and the spectra reacquired until the desired state was achieved. In this way, two additions of triethylamine were alternated with two additions of $HClO_4$ for a total of 8 further additions each of triethylamine/$HClO_4$ (10 total).

S6. pK_a calculation methodology

The pK_a values were obtained using COSMO-RS[16–18] method with empirical corrections. The geometry optimizations were carried out at BP86/TZVP level of theory with RI approximation and applying the COSMO model. They were followed by single-point calculations at BP86/TZVPD level of theory with COSMO model and *Fine* cavity parameter. For every acid and anion, a number of initial geometries were created to identify stable conformers. The vibrational frequency analysis was carried out to confirm that the conformers correspond to local minima (evidenced by absence of imaginary frequencies in the calculated vibrational spectra). The quantum-chemical calculations were carried out with the Turbomole V6.5[19] software package.

COSMOtherm[20] software with parametrization BP_TZVPD_FINE_20 was used to obtain pK_a values in MeCN from the results of DFT calculations (energies, geometries, charge distributions on the molecular surface). All stable conformers were taken into account, weighted by their energies in MeCN.

According to our experience, pK_a values from COSMO-RS calculations usually correlate well with experimental pK_a values (especially within the groups of structurally related compounds), but systematic errors are often present. The available high-quality experimental pK_a data in MeCN (mainly from ref. 21[21]) was used to evaluate the accuracy of calculations for each acid type and to correct systematic errors (see Fig. S4). The uncertainty estimates of the corrected results were derived from the quality of the respective correlation between experimental and calculated data (standard error of regression) and consistency of the results obtained by different correction approaches. The results are detailed in Table S1.

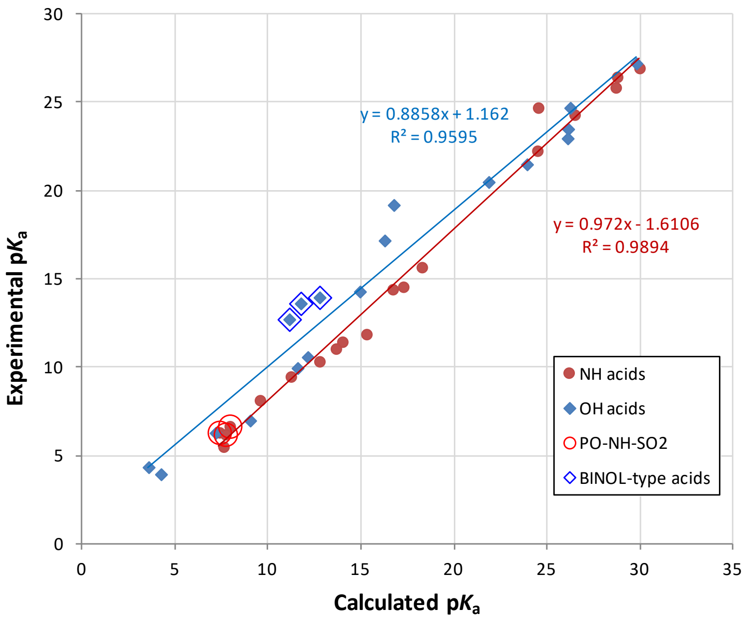

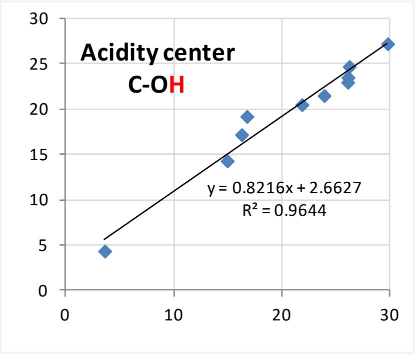

Figure S4: Correlations between experimental and calculated pK_a values of the reference acids.

Table S1: Calculated and corrected pK_a data of the acids of interest, together with standard uncertainty estimates (u).

Acid	Acidity center	Calc. pK_a	Correction method	Corrected pK_a	*u*	Assigned pK_a
**2**	C-OH	24.2	correlation incl. all OH acids	22.6	1.5	**22.6 ± 1.5**
			correlation incl. C-OH acids	22.6	1.3	
**4**	P-OH	16.3	correlation incl. all OH acids	15.6	1.5	**17.0 ± 1.5**
			additive correction for BINOL-type acids	17.9	0.3	
**5**	PO-NH-SO_2	10.0	correlation incl. all NH acids	8.2	0.8	**8.5 ± 0.8**
			additive correction for PO-NH-SO_2 acids	8.8	0.2	
**6**	NH	31.6	correlation incl. all NH acids	29.1	0.8	**29 ± 1**
**7**	NH	26.9	correlation incl. all NH acids	24.5	0.8	**24.5 ± 1.0**

S7. References

(1) Adhikari, S.; Mandal, S.; Ghosh, A.; Das, P.; Das, D. Strategically Modified Rhodamine-Quinoline Conjugate as a CHEF-Assisted FRET Probe for Au3+: DFT and Living Cell Imaging Studies. *J. Org. Chem.* **2015**, *80*, 8530–8538.

(2) Ruíz-Pérez, K. M.; Quiroz-García, B.; Hernández-Rodríguez, M. Prolinamides of Aminouracils, Organocatalyst Modifiable by Complementary Modules. *Eur. J. Org. Chem.* **2018**, *2018*, 5763–5772.

(3) Voituriez, A.; Charette, A. B. Enantioselective Cyclopropanation with TADDOL-Derived Phosphate Ligands. *Adv. Synth. Catal.* **2006**, *348*, 2363–2370.

(4) Beck, A. K.; Gysi, P.; La Vecchia, L.; Seebach, D. (4R,5R)-2,2-DIMETHYL-a,a,a',a'-TETRA(NAPHTH-2-YL)-1,3-DIOXOLANE-4,5-DIMETHANOL FROM DIMETHYL TARTRATE AND 2-NAPHTHYL-MAGNESIUM BROMIDE. *Org. Synth.* **1999**, *76*, 12.

(5) Seebach, D.; Beck, A. K.; Imwinkelzied, R.; Roggo, S.; Wonnacott, A. Chirale Alkoxytitan(IV)-Komplexe Für Enantioselektive Nucleophile Additionen an Aldehyde Und Als Lewis-Säuren in Diels-Alder-Reaktionen. *Helv. Chim. Acta* **1987**, *70*, 954–974.

(6) Pichota, A.; Gramlich, V.; Bichsel, H. U.; Styner, T.; Knöpfel, T.; Wünsch, R.; Hintermann, T.; Schweizer, W. B.; Beck, A. K.; Seebach, D. Preparation and Characterization of New C 2- and C 1-Symmetric Nitrogen, Oxygen, Phosphorous, and Sulfur Derivatives and Analogs of TADDOL. Part II. *Helv. Chim. Acta* **2012**, *95*, 1273–1302.

(7) Seebach, D.; Beck, A. K.; Bichsel, H.-U.; Pichota, A.; Sparr, C.; Wünsch, R.; Schweizer, W. B. Preparation and Characterization of New C2- and C1-Symmetric Nitrogen, Oxygen, Phosphorous, and Sulfur Derivatives and Analogs of TADDOL. Part III. *Helv. Chim. Acta* **2012**, *95*, 1303–1324.

(8) Kang, I. J.; Hsu, S. J.; Yang, H. Y.; Yeh, T. K.; Lee, C. C.; Lee, Y. C.; Tian, Y. W.; Song, J. S.; Hsu, T. A.; Chao, Y. S.; Yueh, A.; Chern, J. H. A Potent, Selective, and Orally Bioavailable HCV NS5A Inhibitor for Treatment of Hepatitis C Virus: (S)-1-((R)-2-(Cyclopropanecarboxamido)-2-Phenylacetyl)-N-(4-Phenylthiazol-2-Yl)Pyrrolidine-2-Carboxamide. *J. Med. Chem.* **2017**, *60*, 228–247.

(9) Palakurthy, N. B.; Dev, D.; Paikaray, S.; Chaudhury, S.; Mandal, B. Synthesis of O-Benzyl Hydroxamates Employing the Sulfonate Esters of N-Hydroxybenzotriazole. *RSC Adv.* **2014**, *4*, 7952.

(10) Thouin, E.; Lubell, W. D. Effective Synthesis of Enantiopure Hydroxamates by Displacement of Resin-Bound Esters with Hydroxylamine. *Tetrahedron Lett.* **2000**, *41*, 457–460.

(11) Fletcher, S. P.; Solà, J.; Holt, D.; Brown, R. A.; Clayden, J. Synthesis of Enantiomerically Enriched (R)-13C-Labelled 2-Aminoisobutyric Acid (Aib) by Conformational Memory in the Alkylation of a Derivative of L-Alanine. *Beilstein J. Org. Chem.* **2011**, *7*, 1304–1309.

(12) Byrne, L.; Solà, J.; Boddaert, T.; Marcelli, T.; Adams, R. W.; Morris, G. A.; Clayden, J. Foldamer-Mediated Remote Stereocontrol: >1,60 Asymmetric Induction. *Angew. Chemie* **2014**, *126*, 155–159.

(13) Zhang, X.; Zhou, Y.; Shen, Y.; Du, L. L.; Chen, J. H.; Leng, Y.; Shen, J. H. Derivatives of (Phenylsulfonamido-Methyl)Nicotine and (Phenylsulfonamido- Methyl)Thiazole as Novel 11β-Hydroxysteroid Dehydrogenase Type 1 Inhibitors: Synthesis and Biological Activities in Vitro. *Acta Pharmacol. Sin.* **2009**, *30*, 1344–1350.

(14) Neilands, J. B. Siderophores: Structure and Function of Microbial Iron Transport Compounds. *Journal of Biological Chemistry*. American Society for Biochemistry and Molecular Biology Inc. November 10, 1995, pp 26723–26726.

(15) Tshepelevitsh, S.; Kütt, A.; Lõkov, M.; Kaljurand, I.; Saame, J.; Heering, A.; Plieger, P. G.; Vianello, R.; Leito, I. On the Basicity of Organic Bases in Different Media. *Eur. J. Org. Chem.* **2019**, No. 40, 6735–6748.

(16) Klamt, A. Conductor-like Screening Model for Real Solvents: A New Approach to the Quantitative Calculation of Solvation Phenomena. *J. Phys. Chem.* **1995**, *99*, 2224–2235.

(17) Klamt, A.; Jonas, V.; Bürger, T.; Lohrenz, J. C. W. Refinement and Parametrization of COSMO-RS. *J. Phys. Chem. A* **1998**, *102*, 5074–5085.

(18) Eckert, F.; Klamt, A. Fast Solvent Screening via Quantum Chemistry: COSMO-RS Approach. *AIChE J.* **2002**, *48*, 369–385.

(19) TURBOMOLE V6.5 2013, a Development of University of Karlsruhe and Forschungszentrum Karlsruhe GmbH, 1989-2007, TURBOMOLE GmbH, since 2007; Available from www.turbomole.com

(20) BIOVIA COSMOtherm, Release 2020; Dassault Systèmes. www.3ds.com

(21) Kütt, A.; Tshepelevitsh, S.; Saame, J.; Lõkov, M.; Kaljurand, I.; Selberg, S.; Leito, I. Strengths of Acids in Acetonitrile. *Eur. J. Org. Chem.* **2021**, *2021*, 1407–1419.
